# Sulforaphane prevents age‐associated cardiac and muscular dysfunction through Nrf2 signaling

**DOI:** 10.1111/acel.13261

**Published:** 2020-10-17

**Authors:** Chhanda Bose, Ines Alves, Preeti Singh, Philip T. Palade, Eugenia Carvalho, Elisabet Børsheim, Se‐Ran Jun, Amrita Cheema, Marjan Boerma, Sanjay Awasthi, Sharda P. Singh

**Affiliations:** ^1^ Division of Hematology & Oncology Department of Internal Medicine Texas Tech University Medical Sciences Center Lubbock TX USA; ^2^ Arkansas Children's Research Institute Little Rock AR USA; ^3^ Center for Neuroscience and Cell Biology University of Coimbra Coimbra Portugal; ^4^ Department of Pharmacology and Toxicology University of Arkansas for Medical Sciences Little Rock AR USA; ^5^ Department of Geriatrics University of Arkansas for Medical Sciences Little Rock AR USA; ^6^ Arkansas Children’s Nutrition Center Department of Pediatrics University of Arkansas for Medical Sciences Little Rock AR USA; ^7^ Department of Biomedical Informatics University of Arkansas for Medical Sciences Little Rock AR USA; ^8^ Departments of Oncology and Biochemistry, Molecular and Cellular Biology Georgetown University Medical Center Washington DC USA; ^9^ Division of Radiation Health Department of Pharmaceutical Sciences University of Arkansas for Medical Sciences Little Rock AR USA; ^10^ Present address: i3S ‐ Institute for Research and Innovation in Health University of Porto Porto Portugal

**Keywords:** cardiac functions, mitochondrial dysfunction, Nrf2, Oxidative stress, sarcopenia, Sulforaphane

## Abstract

Age‐associated mitochondrial dysfunction and oxidative damage are primary causes for multiple health problems including sarcopenia and cardiovascular disease (CVD). Though the role of Nrf2, a transcription factor that regulates cytoprotective gene expression, in myopathy remains poorly defined, it has shown beneficial properties in both sarcopenia and CVD. Sulforaphane (SFN), a natural compound Nrf2‐related activator of cytoprotective genes, provides protection in several disease states including CVD and is in various stages of clinical trials, from cancer prevention to reducing insulin resistance. This study aimed to determine whether SFN may prevent age‐related loss of function in the heart and skeletal muscle. Cohorts of 2‐month‐old and 21‐ to 22‐month‐old mice were administered regular rodent diet or diet supplemented with SFN for 12 weeks. At the completion of the study, skeletal muscle and heart function, mitochondrial function, and Nrf2 activity were measured. Our studies revealed a significant drop in Nrf2 activity and mitochondrial functions, together with a loss of skeletal muscle and cardiac function in the old control mice compared to the younger age group. In the old mice, SFN restored Nrf2 activity, mitochondrial function, cardiac function, exercise capacity, glucose tolerance, and activation/differentiation of skeletal muscle satellite cells. Our results suggest that the age‐associated decline in Nrf2 signaling activity and the associated mitochondrial dysfunction might be implicated in the development of age‐related disease processes. Therefore, the restoration of Nrf2 activity and endogenous cytoprotective mechanisms by SFN may be a safe and effective strategy to protect against muscle and heart dysfunction due to aging.

## Graphical abstract

**Scheme depicts hypothesized decreases of heart and SKM function during aging via ROS and partial reversal by SFN activation of Nrf2 that results in significant restoration of function of both types of muscle.** A decline in heart and skeletal muscle function was observed in aged mice, with altered mitochondrial structure and gene expression, accompnied by decreases in mitochondrial complex activity, Nrf2 binding to antioxidant‐responsive DNA elements and physical endurance. The addition of sulforaphane (SFN) to the diet improved these age‐related changes in older mice to levels observed in younger ones. We demonstrated in this paper that SFN alleviates age‐associated oxidative damage and improves mitochondrial and cardiac function as well as physical endurance in old mice.

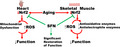

## 1 INTRODUCTION

Aging is a complex biological process regulated by both intrinsic and extrinsic factors. The mechanisms of aging include the actions of reactive oxygen species (ROS), telomere shortening, and hormonal changes. Age‐associated mitochondrial dysfunction and oxidative damage are primary causes for multiple health problems including sarcopenia and cardiovascular disease (CVD) (Miller et al., 2012). Several chemically and functionally diverse scavengers of ROS and antioxidant products have been evaluated for the ability to mitigate age‐induced muscle loss (Barrera et al., 2018), but with little success. The major reasons for their lack of benefit may include low bioavailability and/or low scavenging efficacy toward oxidants and electrophiles in a cellular system. Therefore, promoting the activity of intrinsic antioxidant and cytoprotective pathways may represent a more effective strategy to counter loss of muscle function in the elderly.

The transcriptional regulation of enzymes (and other proteins) that are critical for the adaptation of cells to oxidative and electrophilic stress is controlled by antioxidant‐responsive DNA elements (AREs), also called electrophilic response elements (Suh et al., 2004). A central regulator of cellular responses to electrophilic stress is the transcription factor Nrf2 (nuclear erythroid‐2‐p45‐related factor‐2), shown to be essential for detoxification gene activity (Itoh et al., 2004), including in mammalian cardiac cells and other components of the cardiovascular system (Dastani et al., 2012). Though the role of Nrf2 in myopathy remains poorly defined, its downregulation has been implicated in both sarcopenia and CVD (Gounder et al., 2012). Under physiological conditions, Nrf2 is bound in the cytosol by its repressor, the Kelch‐like ECH‐associating protein 1 (Keap1). Keap1 regulates degradation of Nrf2 in response to electrophiles (Itoh et al., 2004). Electrophiles, including the natural compound sulforaphane (SFN), activate antioxidant and cytoprotective pathways through thiol modification of Keap1, causing it to dissociate from Nrf2 in the cytoplasm. Upon release, Nrf2 accumulates in nuclei, heterodimerizes with a small Maf protein, and activates the transcription of target genes through their AREs (Cao et al., 2006). Nrf2 target genes encode proteins involved in cytoprotective responses, including cellular antioxidant and anti‐electrophile activities as well as biosynthesis of glutathione (GSH), an important cellular antioxidant. To date, all known genes encoding enzymes in electrophile metabolism as well as numerous antioxidant enzymes are regulated at the basal expression level and frequently are inducible by Nrf2 (Dinkova‐Kostova & Abramov, 2015).

Defective mitochondria that generate excessive ROS are detrimental to cells and are frequently cleared by the autophagy/mitophagy pathway, followed by induction of mitochondrial biogenesis in an attempt to restore mitochondrial mass and function (Dinkova‐Kostova & Abramov, 2015) Several recent findings suggest a role of Nrf2 in preserving mitochondrial morphology and integrity during oxidative stress through a direct activation of protective mechanisms (Dinkova‐Kostova & Abramov, 2015).

SFN, a phytochemical in cruciferous vegetables, is a non‐toxic compound recognized for its anti‐aging, anti‐cancer, anti‐diabetic, antimicrobial, and chemopreventive activity in different animal models of disease (Kensler et al., 2013). Enhanced Nrf2 signaling and consequent cytoprotective gene activation is considered the prime mechanism of SFN action (Bai et al., 2013). SFN has shown potential in reducing microglial mediated neuroinflammation and can ameliorate neurobehavioral deficits and reduce the αβ burden in Alzheimer's disease model mice (Uddin et al., 2020). SFN improves muscle function, pathology, protects dystrophic muscle, and alleviates muscle inflammation (Sun et al., 2015). SFN also inhibits dexamethasone‐induced muscle atrophy in myotubes via Akt/Foxo signaling (Son et al., 2017). Moreover, SFN repairs vascular smooth muscle cell dysfunction in age‐related cardiovascular diseases and protects against skin aging through promoting the antioxidant machinery (Sedlak et al., 2018). Currently, several ongoing preclinical and clinical trials study its effect on cancers, insulin resistance, schizophrenia, and autism (Kensler et al., 2013; K. Singh et al., 2014).

Because SFN is known to activate Nrf2 and alleviate oxidant injury, in this study we examined whether treatment with SFN can restore Nrf2 activity, mitochondrial function, and skeletal muscle and heart function of old mice. We also evaluated transcriptome alteration in skeletal muscle and heart of SFN‐treated and control mice. In this study, we established a basis for upregulating Nrf2 activity as a novel therapeutic strategy to mitigate age‐induced muscle and cardiac dysfunction.

## 2 METHODS

This study conformed to the Guide for the Care and Use of Laboratory Animals of the National Institutes of Health, and the work was performed in accordance with a protocol (IACUC#646767‐5) approved by the Central Arkansas Veterans Healthcare System Institutional Animal Care and Use Committee. Two‐month‐old (young) and 21‐ to 22‐month‐old (old) male C57BL/6 mice were obtained from aged rodent colonies of the National Institutes of Health (Bethesda, MD 20892). Mice were housed in the Veterinary Medical Unit at the Central Arkansas Veterans Healthcare System in Little Rock, Arkansas. A maximum of 4 mice per cage were housed together, and each mouse was individually identified by ear punch. The animals had free access to water and diet and were maintained on a 12‐hour dark/12‐hour light cycle. Food and water consumption and body weight of mice were recorded weekly.

### 2.1 Animal treatment protocol for the study

All analyses were performed on 4 groups (n = 10/group). Starting at the age of 2 months and at the age of 21‐22 months, mice were fed with either TD 96163 (Teklad, Madison, WI) control diet (n = 20/control groups) or TD 96163 diet supplemented with SFN (442.5 mg/kg body weight; n = 20/treated groups) for 12 weeks. D,L‐sulforaphane (SFN) was obtained from Toronto Research Chemicals, North York, ON. The experiment was repeated with the same number of animals under similar conditions to test reproducibility of certain experiments.

### 2.2 Intraperitoneal glucose tolerance test (IPGTT)

The test was performed at the completion of SFN diet administration, according to a procedure described by Carvalho et al. (2005). Mice were fasted overnight for approximately 10‐12 hrs by transferring them to clean cages with access to drinking water. The mice received an intraperitoneal injection of glucose (2 g/kg body weight). Immediately before, and 15, 30, 60, 120 min after glucose injection, mice were briefly anaesthetized using isoflurane, and ~5 µl blood was sampled from the tail vein. Glucose concentration was determined using the hand‐held glucometer Alpha‐TRAK 2 Veterinary Blood Glucose Monitoring Meter Kit (Abbott Laboratories). At the end of the test, the mice were returned to their home cages with food and water available *ad libitum*.

### 2.3 Skeletal muscle (SKM) function test

The test was performed at the completion of the SFN diet administration, according to the procedure described by Handschin et al. (2007). Mice were trained and allowed to run on a motorized, speed‐controlled, modular treadmill system, Exer 3/6 (Columbus Instruments, OH). The treadmill was equipped with an electric shock stimulus (set at 1 Hz, 20% output) and an adjustable inclination angle. All mice were acclimatized to the treadmill (first with the belt unmoving, followed by shock grids off but the belt motor turned on) for 15 min/day for three consecutive days followed by 10 min at 8 m/min for three consecutive days before an experiment. For the exercise tolerance test, mice were allowed to warm up at 8 m/min and 0% incline for 5 min. At every 2‐min interval, workload was augmented by alternating increases in belt speed of 2 m/min or increases in incline of 10% grade until mice developed exhaustion or a maximum speed of 46 m/min was reached. Exhaustion was defined when mice were unable to avoid repetitive electrical shocks for 5 continuous seconds. Running time until exhaustion was measured, and the running distance, work, and power were calculated. Distance is a function of time and speed of the treadmill. Work (kJ) was calculated as the product of bodyweight (kg), gravity (9.81 m/s^2^), vertical speed (m/s × angle), and time (s).

### 2.4 Strength test

Kondziela's inverted screen test of forearm isometric muscle strength was performed at the completion of SFN diet administration, according to the procedure described by Au‐Deacon (Au ‐ Deacon, 2013). The mice were placed in the center of a wire mesh screen, and the screen was rotated to an inverted position over 2 sec with the mouse's head declining first. The screen was held steadily 40‐50 cm above a padded surface. The time until the mouse fell off was noted, or the mouse was removed when the criterion time of 60 s was reached.

### 2.5 Immunostaining of PAX7/MAYOD

At the completion of 12 weeks of SFN treatment, all mice were euthanized and the extensor digitorum longus (EDL) (Rosenblatt et al., 1995) was obtained from the lower hindlimb. Myofiber clusters were placed in Matrigel (1 mg/ml) coated 24‐well plates and the cultures were maintained at 37°C in 5% CO_2_ for up to 72 h, with images obtained at 0, 48, and 72 h. Myofibers and satellite cells were fixed in 4% PFA/PBS, permeabilized with 0.5% Triton X‐100/PBS, and blocked using 20% goat serum/PBS. Myofibers were incubated with the primary anti‐MyoD1 (clone 5.8a; DakoCytomation), and the anti‐Pax7 (DSHB) antibodies. Antibody binding was visualized with fluorochrome‐conjugated secondary antibodies (Molecular Probes) before mounting in Faramount fluorescent mounting medium (DakoCytomation) containing 100 ng/ml DAPI. Immunostained myofibers were viewed on an epifluorescence microscope (Nikon E1000, Nikon). The number of cells in each category (i.e., Pax7+ve/MyoD+ve, Pax7−ve/MyoD+ve, or Pax7+ve/MyoD−ve) was counted and expressed as a percentage of the total immunostained cells on the myofiber, and the data from multiple myofibers were pooled to give a population mean. The absolute number of satellite cells per myofiber was determined using unquenched DAPI fluorescence after X‐Gal incubation.

### 2.6 Echocardiography

Prior to euthanasia, control and SFN‐treated mice were subjected to cardiac analysis by echocardiography at the completion of diet administration, using the Vevo 770 instrument (FUJIFILM VisualSonics). Each mouse was anesthetized with 1‐2.5% isoflurane, and hair was removed from the thorax with depilatory cream. Echocardiographs were obtained in the short axis M‐mode at the mid‐left ventricular level to calculate common parameters of systolic function such as ejection fraction and cardiac output (Boerma et al., 2016; Bose et al., 2018; P. Singh et al., 2015).

### 2.7 Oxygraph

At the completion of the 12 weeks of SFN treatment, all mice were euthanized, SKM and heart tissue were obtained for high‐resolution respirometry (HRR) using the Oxygraph‐2k (Oroboros Instruments, Austria) measurements. A section of myocardial tissue (left ventricle) maintaining the orientation of heart along the septum and SKM (gastrocnemius lateralis) were placed in relaxing solution on ice. The relaxing solution (BIOPS) contained 2.77 mM CaK_2_EGTA, 20 mM imidazole, 20 mM taurine, 7.23 mM K_2_EGTA (free Ca^2+^ concentration 0.1 µM), 6.56 mM MgCl_2_, 5.77 mM ATP, 15 mM phosphocreatine, 0.5 mM dithiothreitol, and 50 mM K‐MES (pH 7.1). Approximately 10 mg tissue sample was used to prepare fiber bundles from each tissue. Tissue was teased apart longitudinally using a blade to increase its surface area. SKM and myocardial fibers were permeabilized by gentle agitation in relaxing solution supplemented with 50 µg/ml saponin for 20 min at 4°C followed by washing in ice‐cold respiration medium/mito‐buffer MiR05 (0.5 mM EGTA, 3 mM MgCl_2_, 60 mM potassium lactobionate, 20 mM taurine, 10 mM KH_2_PO_4_, 20 mM HEPES, 110 mM sucrose, and 1 mg/ml essential fatty acid‐free bovine serum albumin (BSA)) (pH 7.1) by agitation for 10 min each at 4°C. Tissue samples weighing ~2 mg (after blotting excess solution on filter paper) were used in duplicate for the respirometric assay.

Mitochondrial complex activities were measured at 37°C by HRR using substrate inhibitor titration protocols. When the chamber oxygenation achieved 400 µM of O_2_, the titration procedure was initiated. The first measured step was fatty acid oxidation, by the addition of 30µl of octanoylcarnitine 0.1 M (final conc. 1.5 mM). Complex I activity was initiated by the addition of 5 µl of 2 M pyruvate, 5 µl of 0.8 M malate, and 10 µl of 2 M glutamate [final conc. 5 mM, 2 mM, and 10 mM, respectively]. To achieve ATP coupled respiration, 20 µl of 0.5 M ADP was added [final conc. 5 mM ADP]. Complex II respiration was stimulated by the addition of 20 µl of 1 M succinate [final conc. 10 mM succinate]. Complex V inhibition was achieved with the addition of 1 µl of 4 mg/ml oligomycin (final conc. 0.2 µg/ml). Then, the chamber was allowed to re‐oxygenate until 400 µM of O_2_, and cytochrome C oxidase (COX) activity was measured following the addition of 5 µl of 5 mM antimycin A, 5 µl of 800 mM ascorbate, and 5 µl of 200 mM N,N,N′,N′‐tetramethyl‐p‐phenylenediamine (TMPD) (final conc. 12.5 µM, 2 mM, and 0.5 mM, respectively). Membrane integrity was evaluated by adding 5 µl of 4 mM cytochrome C (final conc. 10 µM). Data acquisition and analysis was performed using DATLAB 4.2 software (Oroboros). Respiratory rates were expressed (pmol/s) per mg of dry tissue weight (Rose et al., 2019).

### 2.8 Immunostaining of electron transport chain (ETC) complex protein and nitrotyrosine adducts in the heart

At the completion of the 12 weeks of SFN treatment, all mice were euthanized, and left ventricular tissue was fixed in 10% phosphate‐buffered formalin and embedded in paraffin. Sections (5 µm) were deparaffinized in ethylene and rehydrated through gradient ethanol (100‐70%), permeabilized by 1% Triton X‐100, and immersed in 3% H_2_O_2_ for 10 min to inactivate endogenous peroxidase activity. The sections were blocked with 5% goat serum/1% BSA in PBS for 30 min at room temperature and then immunostained with primary antibodies against Ndusf3 (1:200; Abcam, ab110246) and CORE‐2 (1:300; Invitrogen, 459220) overnight. Analysis of the stained tissue sections was performed with light microscopy (Nikon E1000, Nikon). Immunohistochemical detection of nitrotyrosine staining was performed using primary antibodies against nitrotyrosine (1:6000 dilution). Immunoreactivity was detected by Dako Envision+System‐HRP. Counterstaining was performed using Mayer's Hematoxylin (Electron Microscopy Science).

### 2.9 Transmission electron microscopy

Left ventricular heart tissue samples were fixed overnight in 2.5% glutaraldehyde/0.05% malachite green in 0.1 M sodium cacodylate buffer. Samples were post‐stained with 1% osmium tetroxide/0.8% potassium hexacyanoferrate (III), 1% tannic acid, and 0.5% uranyl acetate followed by dehydration in a graded alcohol series and propylene oxide and embedded in Araldite‐Embed 812 (Electron Microscopy Sciences). Sections of 50 nm were collected on a Leica UC7 microtome and post‐stained with uranyl acetate and lead citrate. Images were collected using a Tecnai F20 (FEI Company, Hillsboro, OR) transmission electron microscope at 80 kv (Bose et al., 2018).

### 2.10 Real‐time polymerase chain reaction

Total RNA was isolated from ventricular heart and SKM (gastrocnemius) tissue samples of control and SFN‐treated mice. cDNA was prepared as described previously (Singh et al., 2015). Real‐time polymerase chain reactions (qPCR) were performed on a DNA Engine Opticon 2 Detection System (MJ Research, Waltham, MA) with the SYBR green Master containing 0.3 µM gene‐specific primers using manufacturer's recommended cycling protocol. Gene expression levels were normalized by calculating for each individual animal the difference, ∆Ct, between ribosomal protein S3 (RPS3) and the gene of interest (Singh et al., 2008). The average ∆Ct for each replicate was exponentially transformed to obtain the expression level (2^–∆Ct^) for each animal (Singh et al., 2015).

### 2.11 Active Nrf2 binding assay

Nuclei were extracted from the left ventricle of the heart and SKM tissues using the Nuclear Extract Kit (Active Motif, Carlsbad, CA) as per manufacturer's instructions. Nrf2 activity was measured in nuclear extracts by an Nrf2‐DNA‐binding ELISA kit (Active Motif) as we have reported before (Singh et al., 2015).

### 2.12 Statistical analyses

All data are presented as mean ±standard deviation (SD) and were analyzed using Prism 8 (GraphPad Software, San Diego, CA, USA). The unpaired Student's *t* test was used when two groups were compared and a one‐way analysis of variance (ANOVA) followed by the Bonferroni or Tukey post hoc test was used when three or more groups were compared. Survival curves were analyzed using the log‐rank test. A *p*‐value < .05 was considered a significant difference between groups.

### 2.13 RESULTS

### 2.14 SFN improved survival but did not affect animal body weight

**FIGURE 1:**
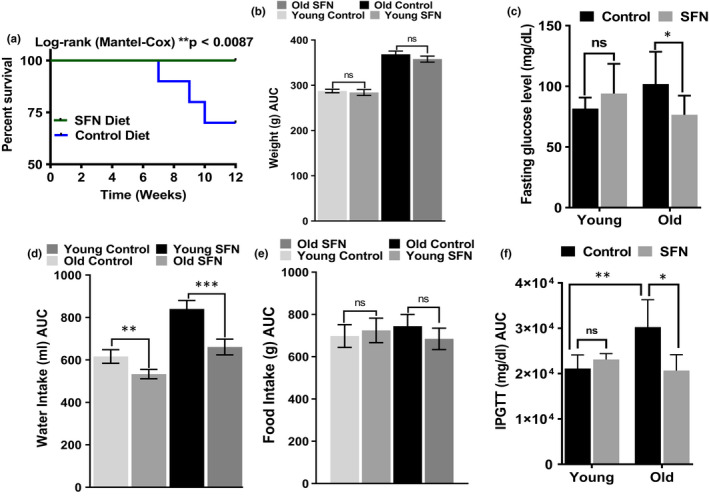
Effects of sulforaphane (SFN) diet on survival, body weight, food intake, water intake, fasting glucose, and glucose tolerance of mouse. Old mice with SFN supplementation improved significantly their survival (a), compared with mice on control diet (*p* = .0087, n = 20) each group. After the start of SFN or control diet, young and old mice were weighed for their body weight (b), water intake (d), and food intake (e), weekly. The data shown represent the means ±SD (n = 10). Statistical significance between SFN or control diet fed mice of the same group was determined using repeated measures two‐way ANOVA followed by Bonferroni test or unpaired Student's *t* test (lower panel). *p < .05 and ns = non‐significant, compared with the same group. SFN also reduced fasting glucose and improved glucose tolerance in old mice challenged with glucose. (c) Fasting blood glucose levels of young and old mice on control or SFN diet were measured after an 8 h fasting period. Means ±SD (n = 10) are shown; the difference between control or SFN diet fed old mice is statistically significant *p < .05 and **p < .01 by a *t* test. (f) Young and old mice on control or SFN diet were fasted for 4 h and were given an intraperitoneal injection of glucose (2 g/kg of body weight). The area under the blood glucose level vs time curve was calculated by numerical integration between 0 and 120 min for each individual mouse, and the mean ±SD (n = 10) of the areas is shown. Differences between young mice are not statistically significant but improve significantly in old mice on SFN diet

At the age of 21‐22 months, male C57BL/6 mice were placed on SFN containing diet or control diet (n = 20 mice per group) for a total of 12 weeks. During the time period of SFN administration, 6 mice from the control group died. On the other hand, all mice in the SFN diet group survived until the end of the experiment at which time they were 24‐25 months old (Figure 1a) Dietary supplementation with SFN did not affect the body weight of either young or old mice. As expected, the body weight of young mice increased more rapidly than old mice in both diet groups (Figure 1b). On the other hand, water intake was affected by supplementation with SFN (Figure 1c). Water intake was significantly lower in young mice fed with the SFN diet compared to the young mice on the control diet. The same was observed in the older animal groups. However, food intake was unaffected (Figure 1d). Furthermore, hematological parameters of young and old mice were not affected by the supplementation of SFN (Table S1).

### 2.15 SFN reduced fasting glucose and improved glucose tolerance in old mice

Fasting blood glucose levels were no higher in young mice on the SFN diet than for the young control mice (Figure 1e). On the other hand, the old mice fed on the SFN diet showed significantly lower glucose levels (mean ±SD: 77 ± 16 mg/dl) compared to old control mice (102 ± 9 mg/dl). In both diet groups, the glucose levels were within or below the normal range for the C57BL/6 strain (Surwit et al., 1988). Because the difference in fasting blood glucose levels between control and treated animals was modest, an intraperitoneal glucose tolerance test was performed. SFN treatment did not affect the response to glucose administration in young animals (Figure 1f). However, in old mice on control diet, blood glucose levels increased more significantly, after glucose injection compared to young animals. Furthermore, the SFN treatment reversed this increase in glycaemia and improved glucose tolerance in the old animals (Figure 1f).

### 2.16 SFN improves exercise capacity in old mice

**FIGURE 2:**
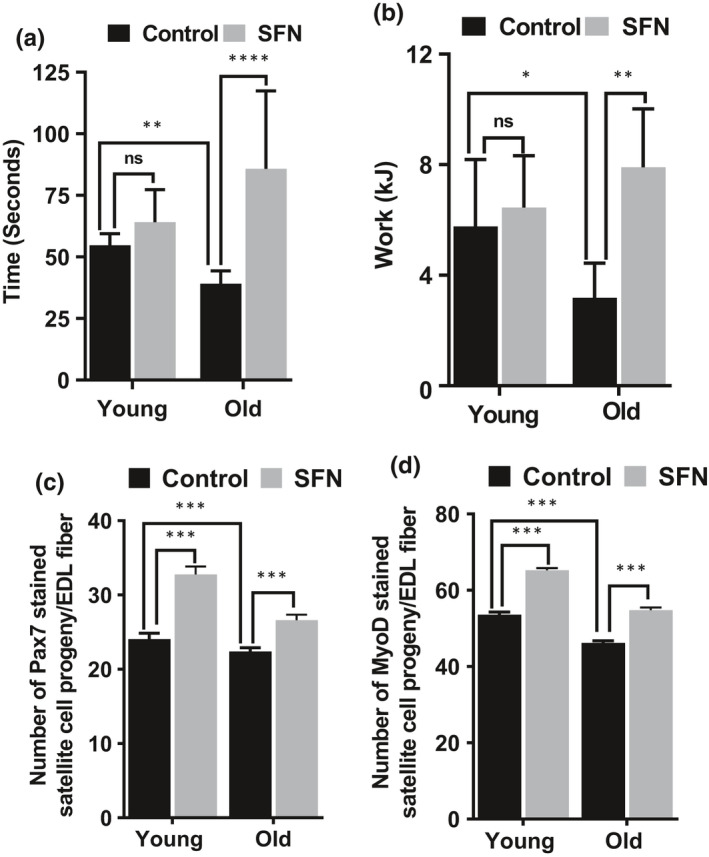
Sulforaphane (SFN) improves grip strength, exercise performance, and numbers of skeletal muscle stem cells in the mouse. (a) Combined forelimbs grip test was used to measure the muscle strength of mice. (b) After acclimatization, mice were made to run on a treadmill with a 10% slope and increasing speed to exhaustion. SFN supplemented diet significantly (*p < .05 and ****p < .0001 by a *t* test) improved exercise capacity and grip strength of old mice (n = 10). Batches of myofibers from the same group were coimmunostained for Pax7 (c) and MyoD (d). Values represent population mean from the pooled data from T72 myofibers from young and old age group supplemented with SFN or control diet alone. The number of satellite cells in each category is expressed as a mean percentage of the total immunostained cells present on the myofiber (***p < .001 by a *t* test, two‐way ANOVA)

We tested whether SFN treatment would improve muscle strength and exercise capacity. For this purpose, we challenged young and old mice, fed with control or SFN diet, with involuntary physical exercise testing of muscle strength. First, we tested them for grip strength by making them hold on to a thick wire. Young and old mice fed with control diet were able to hold on for an average of 54 ± 5 and 39 ± 5 s, respectively. Young and old mice on SFN diet were able to hold on for longer periods of time, 64 ± 13 and 86 ± 12 s, respectively (Figure 2a). Surprisingly, the old mice fed SFN were able to hold on longer than the young mice fed SFN. In addition, we compared the exercise capacity of control and SFN‐treated young and old mice by having them run on a motorized, speed‐controlled, modular treadmill system (Handschin et al., 2007). Old mice on control diet had a lower exercise capacity compared to their young counterparts (Figure 2b). Feeding with an SFN‐rich diet resulted in a significantly improved exercise capacity in the old mice. The SFN‐fed old mice performed similarly to young animals on the treadmill.

### 2.17 SFN increased the numbers of skeletal muscle stem cells and their function

Myofibers isolated from the EDL muscles of the lower hindlimb were cultured for 72 h. The satellite cell‐derived myoblasts were fixed and immunostained for Pax7 and MyoD as indicators of satellite cell proliferation and differentiation (Figure S1‐S8). A large portion of the satellite cells expressed Pax7 and even more expressed MyoD (Figure 2c). Expression of Pax7 in SKM stem cells does not affect MyoD expression and contributes to the growth and regeneration of the SKM (Zammit et al., 2006). The proportions of the Pax7‐ (Figure 2c) and MyoD (Figure 2d)‐positive satellite cell progeny in both young and old mice on SFN diet were significantly higher than in their age‐matched controls. SFN treatment may increase new satellite cell formation to meet the routine needs of muscle homeostasis, or potentially the more sporadic demands for hypertrophy or repair.

### 2.18 SFN decreased markers of SKM aging, oxidation, and apoptosis

Histology revealed more of the cross‐sectional area in slides was occupied by myofibers after 2 months on SFN diet (Figure S9). Similarly, there was less staining for muscle myostatin, a negative modulator of SKM mass (Figure S10). Finally, we found evidence that SFN reduced 8OHdG, a marker of oxidation (Figure S11) and Tunel staining, a surrogate marker for apoptosis (Figure S12).

### 2.19 SFN treatment of old mice also improves cardiac function

**FIGURE 3:**
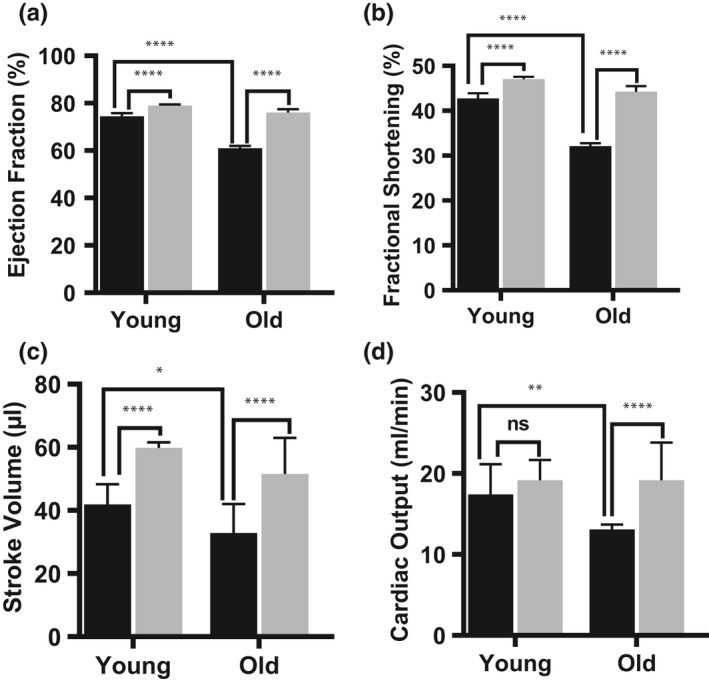
Sulforaphane (SFN) treatment protects mouse from age‐associated cardiomyopathy. Preservation of cardiac function was assessed by evaluating: (a) ejection fraction, (b) fractional shortening, (c) stroke volume, and (d) cardiac output, which were significantly preserved in old mice fed with SFN supplemented diet. Black bar represents animals fed on control diet and gray bar represents animals fed on SFN supplemented diet. Statistical significance ***p < .001 and ****p < .0001 was determined by ANOVA used followed by Tukey (n = 10)

A decrease in cardiac/respiratory function is known to limit exercise capacity in the elderly (Farkhooy et al., 2018). Cardiac aging is an intrinsic process with profound cellular and molecular changes that results in impaired cardiac function (Vigorito & Giallauria, 2014). We examined whether SFN restored cardiac function in old mice. Old mice on control diet showed reduced ejection fraction (61.0 ± 1.0%), fractional shortening (32.1 ± 0.7%), and stroke volume (32.8 ± 9.2 µl) compared to young mice on control diet (Figure 3a‐c). On the other hand, SFN supplementation improved the ejection fraction (76.0 ± 1.4), fractional shortening (44.2 ± 1.3%), and the stroke volume (51.6 ± 11.3 µl) in the old mice. While SFN also significantly increased these parameters in young mice, the effect on ejection fraction and fractional shortening was larger in the old mice. There was no significant effect of SFN supplementation on cardiac output in young mice, while cardiac output in old mice was significantly improved (Figure 3d). As a result, cardiac output in SFN‐fed old mice was similar to that of young controls. We conclude that SFN‐fed old mice developed resistance to age‐associated loss of cardiac function.

### 2.20 SFN protects ultrastructure of cardiac mitochondria in aging heart

**FIGURE 4:**
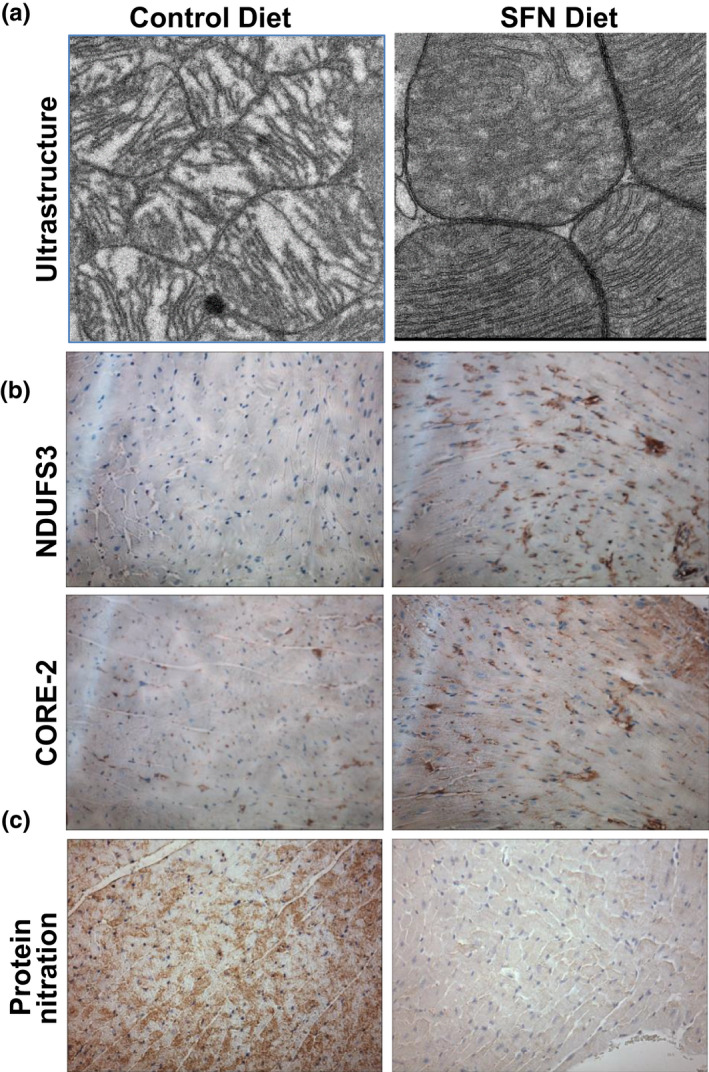
Sulforaphane (SFN) increases ETC complex protein expression and protects ultrastructure of cardiac mitochondria in aging heart. (a) SFN prevents mitochondrial cristae disarrangement, partial cristolysis, and electron‐lucent matrix in aging heart (representative of n = 3). (b) Immunohistochemical detection of NDUSF3 and CORE‐2 (brown staining) showed increased levels in left ventricle of SFN‐fed old mice (representative of n = 5). (c) SFN supplementation reverses protein nitration from oxidative stress in aging heart (representative of n = 5)

Mitochondria, the primary energy source for high energy demanding cardiomyocytes, occupy over 40% of their cell volume (~5,000 mitochondria/cardiomyocyte). Cardiomyocyte function is critically dependent on the health of these organelles (Strom et al., 2016). Additionally, age‐associated mitochondrial dysfunction can be caused by changes in mitochondrial ultrastructure due to oxidative damage (Vays et al., 2014). To determine whether SFN protects against age‐associated changes in cardiac mitochondrial ultrastructure, we compared mitochondrial morphology between hearts from control and SFN‐fed old mice using transmission electron microscopy. The results suggest that SFN protects mitochondria against age‐associated cristae disarrangement, partial cristolysis, and reduced electron density of the matrix (Figure 4a). Since the enzymes involved in oxidative phosphorylation are located on the inner mitochondrial membrane, the surface area and number of cristae are generally correlated with the metabolic activity exhibited by a cell (Arismendi‐Morillo, 2011). To further verify the increase/protection in electron transport chain (ETC) complex activity observed in the hearts of aging mice treated with SFN, we performed immunostaining with antibodies against proteins from complex I (NDUFS3‐a nuclear DNA‐encoded subunit) and complex III (CORE2‐a mitochondrially encoded subunit). Mitochondrial complex I & III subunit protein expression was increased in the aging heart after SFN treatment compared to the heart of age‐matched untreated animals (Figure 4b). These data suggest that one mechanism by which SFN may maintain mitochondrial function is by inducing mitochondrial protein expression. Alternatively, the decreased protein expression found in cardiomyocyte mitochondria in the old mice might be due to oxidative damage. Immunohistochemical analysis of left ventricular tissues revealed that SFN‐fed old mice had lower levels of nitrotyrosine protein adducts (a marker of oxidative stress) (Figure 4c). These effects may have contributed to increased autophagy in hearts of old mice and to the mitigation of that autophagy by SFN, as illustrated by the appearance of LC3‐II in old mice and its disappearance after treatment with SFN (Figure S13).

### 2.21 SFN improves mitochondrial function in the old mice

**FIGURE 5:**
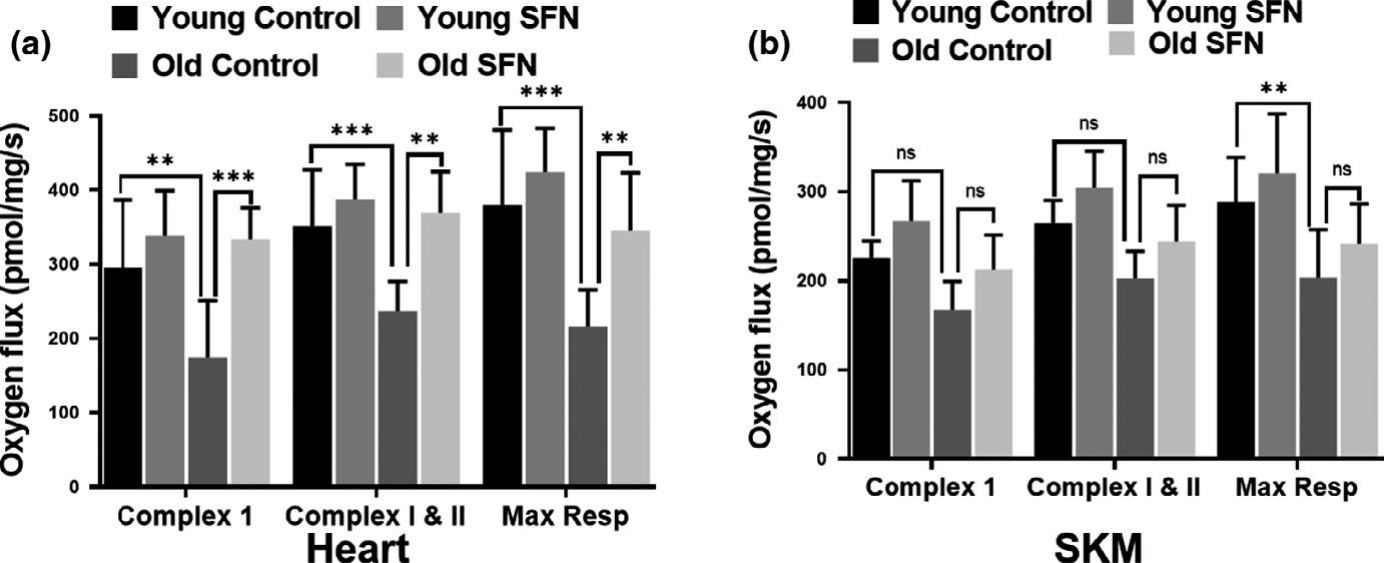
Sulforaphane (SFN) improves ETC function in aging heart. Respiration status of complex I, II+III, and maximum respiration of the ETC was evaluated in fresh heart (a) and SKM biopsies (b) from young and old mice (n = 10) fed with i) control diet or ii) SFN supplemented diet (442.5 mg per kg diet; 3 months), using the substrate inhibitor titration protocol as described in Methods section. According to oxygen flux measures, old mice fed with SFN show improved complex I, I+II and maximum respiration compared to control diet fed old mice. Each bar represents mean ±SD (n = 10); statistical significance was evaluated by performing an unpaired *t* test (ns > 0.05, **p < .01 and ***p < .001 by a *t* test)

Heart tissue is particularly rich in mitochondria to meet its high metabolic demand. Disruption of mitochondrial respiratory complexes can lead to the generation of oxidants, ATP depletion, and cardiomyocyte malfunction. Mitochondrial function has long been recognized to decline during aging, resulting in increased oxidative stress (Hebert et al., 2015). We measured cardiac and SKM tissue mitochondrial function by high‐resolution respirometry. ETC activity was ≈30% lower in the old compared to the young mouse hearts, and SFN protected the ETC from this age‐associated decline in oxygen flux (Figure 5a). Additionally, the activities of the ETC complexes I, I+II, and maximum respiration were increased in SFN‐fed animals, but this increase was significant only in the old mice. Similar trends were seen in mitochondria from the SKM of SFN‐fed animals, but these increases did not reach the level of significance (Figure 5b).

### 2.22 SFN restores Nrf2 activity in the heart and SKM of old mice

**FIGURE 6:**
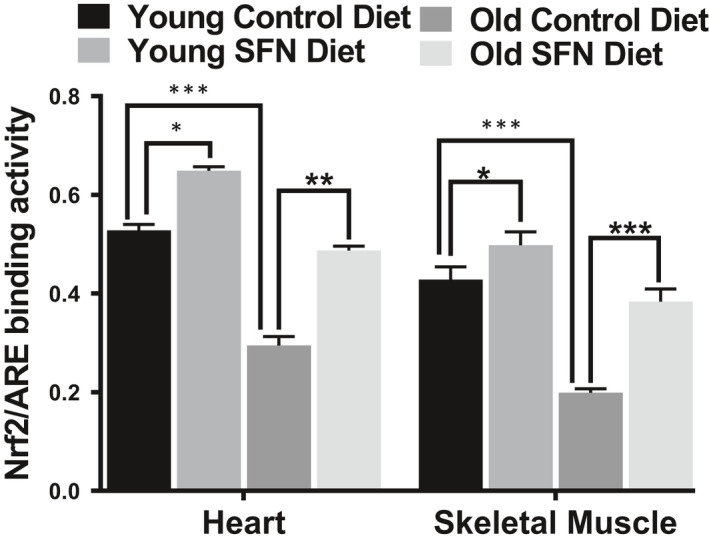
Sulforaphane (SFN) supplemented diet improves Nrf2 activity. SFN supplementation improves Nrf2‐ARE‐binding activity from old mouse heart (n = 5). Statistical significance *p < .05, **p < .01 and ***p < .001 was determined by ANOVA followed by Tukey's multiple comparison test.

Aside from effect on mitochondria, an age‐associated decline in Nrf2 function has been well documented (Gounder et al., 2012), but it is not clear if SFN can restore Nrf2 activity in the older population. We recently reported that Nrf2 activity increases with SFN treatment and is key to protecting the heart from oxidant injuries such as doxorubicin and ionizing radiation (Boerma et al., 2015; P. Singh et al., 2015). Therefore, we compared Nrf2 ARE‐binding activity in the hearts and SKM of young and old mice. Nrf2 activity was upregulated in the nuclear extracts of both hearts and SKM of SFN‐fed young and old mice compared to their age‐matched controls (Figure 6).

### 2.23 Results on heart and skeletal muscle qRT‐PCR to further delineate potential mechanisms by which SFN works

To further elucidate the mechanisms of SFN‐mediated enhancement of aging cardiac and SKM functions in mice, we examined the expression of a number of genes related to oxidant and electrophile metabolism. The selected genes are involved in antioxidant, anti‐electrophile activity, mammalian longevity, and glutathione synthesis and loss (Tables 1 and 2), all known to be regulated by Nrf2 (Hayes & Dinkova‐Kostova, 2014). We examined cardiac and SKM transcript levels of *catalase*, *Sod1*, *Sod2*, *HO1*,*Pxdn*, *Gpx1*, *HO‐1, Gsta4, Akr3, Akr7, Akr 8, Sirt1*, *Pgc1*, *Gclc*, *Gclm*, and *Nrf2* (See Table 1 for full names of the genes). *In silico* or *in vitro* analysis of the *Gclc*,*Gclm*,*Gsta4*,*Nqo*‐*1*,*Ho*‐*1*, and *Sod2* promoter regions have revealed consensus ARE‐binding sites for Nrf2 (Tonelli et al., 2018).

**TABLE 1:** Relative abundance of Nrf2 and Nrf2 target gene transcripts from **hearts** of control young and old mice and those fed SFN

	Young control	Young + SFN	Old control	Old + SFN
Genes for antioxidant enzymes				
*Cat*	0.45 ± 0.05	1.36 ± 0.20****	0.14 ± 0.01^x^	0.41 ± 0.03*
*Sod1*	1.92 ± 0.13	3.75 ± 0.27****	0.54 ± 0.18^xxxx^	1.61 ± 0.16****
*Sod2*	1.77 ± 0.09	6.20 ± 0.42****	0.65 ± 0.09^xxxx^	2.20 ± 0.06****
*Pxdn*	0.12 ± 0.03	0.94 ± 0.15****	0.13 ± 0.04^ns^	0.35 ± 0.15^ns^
*Gpx1*	0.53 ± 0.18	3.18 ± 0.51****	0.53 ± 0.12 ^ns^	2.82 ± 0.38****
Genes for enzymes associated with aging				
*HO−1*	0.01 ± 0.01	0.02 ± 0.01^ns^	0.01 ± 0.01^ns^	0.01 ± 0.01^ns^
*Sirt1*	0.27 ± 0.10	0.41 ± 0.15^ns^	0.04 ± 0.02^ns^	0.25 ± 0.13^ns^
*Pgc1*	0.08 ± 0.04	0.23 ± 0.08^ns^	0.04 ± 0.10^ns^	0.09 ± 0.02^ns^
Genes for anti‐electrophile enzymes				
*Gsta4*	1.30 ± 0.24	5.20 ± 0.60****	1.38 ± 0.50 ^ns^	4.59 ± 1.06****
*Akr3*	0.41 ± 0.03	0.66 ± 0.19^ns^	1.70 ± 0.17^xxxx^	1.16 ± 0.21**
*Akr7*	0.016 ± 0.05	0.016 ± 0.09^ns^	0.001 ± 0.01^xxxx^	0.001 ± 0.01 ^ns^
*Akr8*	0.016 ± 0.05	0.08 ± 0.03^ns^	0.16 ± 0.09^ns^	0.29 ± 0.27^ns^
Genes for glutathione synthesis and loss				
*Gclc*	0.13 ± 0.02	0.17 ± 0.01****	0.06 ± 0.02^*^	0.10 ± 0.02^ns^
*Gclm*	0.06 ± 0.01	0.05 ± 0.01^ns^	0.04 ± 0.02^ns^	0.05 ± 0.03^ns^
Gene for major regulator of antioxidant and cellular protective genes				
*Nrf2*	0.35 ± 0.04	2.12 ± 0.50****	0.17 ± 0.06^x^	0.67 ± 0.13**

Relative levels of transcripts encoding Nrf2, Nrf2‐driven antioxidant, and anti‐electrophile enzymes, enzymes associated with aging, and enzymes involved in glutathione synthesis and loss were measured by qRT‐PCR in young and old mice hearts of control and treated animals. Gene expression levels were normalized to the S3 ribosomal protein transcript by calculating for each individual animal the difference, ∆Ct, in the respective cycle numbers. The ratio of the gene expression level was calculated by the ∆∆Ct method as described in Methods section, and values are shown as mean±SD (n = 5). Asterisks denote a statistical significance by Tukey's post hoc after two‐way ANOVA of all groups difference (^*^ *p* < .05,^**^ *p* < .01,^***^ *p* < .001,^****^ *p* < .05, two‐tailed *t* test) between control and treated (young or old) group of animals. “x” denotes similar comparisons between untreated young and old groups of animals (^xx^ *p* < .01,^xxxx^ *p *< .0001, two‐tailed *t* test). “ns” denotes *p* > .05 in all comparisons.

Abbreviations: Akr 3, 7 and 8, aldo‐keto reductases 3, 7, and 8; Cat, catalase; Gclc, glutamate‐cysteine ligase catalytic subunit; Gclm, glutamate‐cysteine ligase modifier subunit; Gpx1, glutathione peroxidase; Gsta4, glutathione‐S‐transferase alpha 4; HO‐1, heme oxygenase 1; Nrf2, nuclear factor erythroid 2‐related factor 2; Pgc1, ppar gamma coactivator 1; Pxdn, peroxidasin, a peroxidase secreted extracellularly; Sirt1, sirtuin 1; Sod1 and Sod2, superoxide dismutases 1 and 2.

**TABLE 2:** Relative abundance of Nrf2 and Nrf2 target gene transcripts from **skeletal muscle** of control young and old mice and those fed SFN

	Young control	Young + SFN	Old control	Old + SFN
Genes for antioxidant enzymes				
*Cat*	1.38 ± 0.48	2.66 ± 0.20****	0.19 ± 0.06^xxxx^	1.64 ± 0.23****
*Sod1*	1.65 ± 0.37	4.42 ± 0.69****	0.98 ± 0.24^xx^	4.35 ± 0.85****
*Sod2*	1.63 ± 0.22	6.48 ± 1.02****	1.18 ± 0.41^ns^	4.45 ± 0.49****
*Pxdn*	0.32 ± 0.08	1.37 ± 0.24****	0.33 ± 0.09^ns^	0.87 ± 0.08*
*Gpx1*	1.71 ± 0.38	4.53 ± 0.27****	0.44 ± 0.08^xxxx^	1.31 ± 0.25***
Genes for enzymes associated with aging				
*HO−1*	0.03 ± 0.01	0.12 ± 0.02 ^ns^	0.01 ± 0.001 ^ns^	0.01 ± 0.001 ^ns^
*Sirt1*	0.30 ± 0.13	1.07 ± 0.20*	0.09 ± 0.04 ^ns^	0.40 ± 0.10 ^ns^
*Pgc1*	0.06 ± 0.04	0.19 ± 0.07^ns^	0.05 ± 0.10 ^ns^	0.08 ± 0.02 ^ns^
Genes for anti‐electrophile enzymes				
*Gsta4*	2.51 ± 0.81	5.71 ± 0.50****	0.90 ± 0.30^xxxx^	2.45 ± 0.18****
*Akr3*	0.03 ± 0.01	0.05 ± 0.02^ns^	0.01 ± 0.001^ns^	0.02 ± 0.001 ^ns^
*Akr7*	0.65 ± 0.24	0.76 ± 0.30^ns^	1.18 ± 0.31*	0.83 ± 0.25^ns^
*Akr8*	0.07 ± 0.02	0.08 ± 0.03 ^ns^	0.03 ± 0.01 ^ns^	0.06 ± 0.05^ns^
Genes for glutathione synthesis and loss				
*Gclc*	0.07 ± 0.05	0.14 ± 0.01*	0.06 ± 0.01^ns^	0.11 ± 0.04*
*Gclm*	0.06 ± 0.01	0.19 ± 0.04*	0.04 ± 0.02^ns^	0.09 ± 0.04*
Gene for major regulator of antioxidant and cellular protective genes				
*Nrf2*	0.90 ± 0.37	1.77 ± 0.82***	0.26 ± 0.07^xx^	0.90 ± 0.40**

Relative levels of transcripts encoding Nrf2, Nrf2‐driven antioxidant and anti‐electrophile enzymes, enzymes associated with aging, and enzymes involved in glutathione synthesis and loss were measured by qRT‐PCR in young and old mice skeletal muscle of control and treated animals. Gene expression levels were normalized to the S3 ribosomal protein transcript by calculating for each individual animal the difference, ∆Ct, in the respective cycle numbers. The ratio of the gene expression level was calculated by the ∆∆Ct method as described in Methods section, and values are shown as mean ± SD (n = 5). Asterisks denote a statistical significance by Tukey's post hoc after two‐way ANOVA of all groups difference (^*^ *p* < .05,^**^ *p* < .01,^***^ *p* < .001,^****^ *p* < .05, two‐tailed *t* test) between control and treated (young or old) group of animals. “x” denotes similar comparisons between untreated young and old groups of animals (^xx^ *p* < .01,^xxxx^ *p* < .0001, two‐tailed *t* test). “ns” denotes *p* > .05 in all comparisons.

Cardiac transcript levels of a number of antioxidant and anti‐electrophile genes were significantly increased in hearts from SFN diet fed young and old mice, as compared to those fed with control diet (Table 1). This shift in metabolism may explain why a diet containing SFN is cardioprotective and enhances exercise capacity. Most of the genes examined were expressed at lower levels in the old control group. The resulting lower level of antioxidant and anti‐electrophilic defense mechanisms in old control versus SFN‐fed animals could contribute to the oxidative damage in the heart. In SFN diet fed animals, levels of certain transcripts, notably *Sod1*, *Sod2*, *Cat*, and *Nrf2*, decreased in hearts from old mice and were essentially restored by SFN in the diet. *Akr3* demonstrated an increase in hearts of old mice and a partial restoration by SFN in the diet.

Table 2 presents results from similar experiments on SKM from the same mice. In this case, even more of these genes were downregulated in the old mice and were largely restored by SFN in the diet: *Cat*,*Sod1*,*Gpx1*,*Gsta4*, and *Nrf2*. Although *Sod2*,*Pxdn*,*Gclc*, and *Gclm* were unaffected by aging, they also were upregulated by SFN in the diet of old mice. Together, these results suggest that the Nrf2 pathway was induced upon SFN treatment in old mice and enhanced protective mechanisms in SKM, possibly even more than in hearts. Together, these results suggest that the Nrf2 pathway was induced upon SFN treatment in the young and old mice and enhanced protective mechanisms.

## 3 DISCUSSION

SFN prolonged life in old mice, decreased their likelihood of becoming diabetic, and increased their exercise capacity. Part of the increase in exercise capacity was certainly due to increased SKM function but some may have been due to preservation of cardiac function. In addition, comparison of survival curves of old mice on SFN or control diet during the study period demonstrates a significant protection from mortality of aged animals on SFN diet (log‐rank (Mantel‐Cox) test, *p* < .0087; Gehan–Breslow–Wilcoxon test, *p* < .0089).

We performed this SFN diet study using young and old C57BL/B6J mice because this strain is relatively long‐lived and has variable causes of death but a low tumor incidence (Treuting et al., 2008). Previous studies have shown that SFN treatment decreases body weight gain and food intake in mice fed on a high‐fat diet or high‐fat high‐sucrose diet fed obese mice and alters metabolic parameters (Shawky et al., 2016; Shawky & Segar, 2018). Using a more normal diet, we found no effects of SFN on body weight or food intake, although we did find a decreased water intake in the old mice fed SFN. We speculate that increased muscle mass may have made up the difference in body weight that reduced water intake should have caused. The mice showed no statistically significant effects on hematological parameters (Table S1). Fasting blood glucose level was slightly lower in old mice on SFN diet than in old control animals, but remained in the published normal range. As seen in Figure 1f, IPGTT is normal in young mice, but insulin resistance became evident in old mice on control diet compared with old mice on SFN diet. The differences between responses of individual older mice (control vs. SFN diet) to insulin suggest that old mice on control diet began to develop metabolic syndrome and pathology. These results suggest that SFN diet may have a beneficial effect on insulin resistance in these old mice.

Findings from the current study include that treadmill exercise capacity and responses to forelimb grip strength significantly improved in old mice fed the SFN diet. Treadmill exercise capacity was approximately 1.5 times higher in old mice fed SFN diet compared with mice fed control diet. When comparing forelimb grip strength, old SFN‐fed mice remained hanging on a wire more than twice as long as old mice on control diet. Performance of old SFN‐fed mice was even better than young mice. Increased Pax7‐ and MyoD‐positive satellite cell progeny in EDL myofibers of SFN‐fed mice suggests an improvement in muscle regeneration in aged mice. Overall, we have demonstrated that the SFN diet intervention improved exercise performance, grip strength, and increased muscle stem cell formation in old mice, suggesting that SFN may be a novel therapeutic approach to reduce sarcopenia in the aging population.

SFN also produced therapeutic effects on heart function in the old mice. Our results suggest that treatment with SFN significantly improves cardiac ejection fraction, fractional shortening, stroke volume, and cardiac output in aged mice, bringing resting cardiac function in old mice to the level of young mice. These particular mice appear to be more prone to systolic dysfunction than most other mouse strains studied, which suffer primarily from diastolic dysfunction (de Lucia et al., 2019). However, there are reports of decreased left ventricular ejection fraction contributing to systolic dysfunction in aging mice (Han et al., 2020; Quarles et al., 2020) and we have previously demonstrated that SFN confers protection from doxorubicin‐induced oxidative stress that causes abnormalities in left ventricle ejection fraction and fractional shortening (Benes et al., 2013; Bose et al., 2018; Singh et al., 2015).

This study has several important limitations. It was performed in male mice only; thus, sex differences are not known. In addition, this study was performed on a relatively small sample size, only utilized two age groups and was of relatively short duration (12 weeks). We have yet to fully delineate the differences in mechanism affected by SFN in SKM versus heart. Finally, the translation of findings to humans is unknown, particularly with respect to heart failure. It is however clear that both skeletal and cardiac muscle function decline in elderly humans and contribute substantially to a decreased quality of life. In this setting, SFN may be a promising strategy to attenuate the aging process in these tissues.

Increased ROS production in the aging heart can lead to necrosis and apoptosis of cardiomyocytes and proliferation of fibroblasts with excess generation of collagen and development of fibrosis, and mitochondrial damage; these events subsequently lead to cardiac remodeling and dysfunction (Dai et al., 2014). The heart, unfortunately, expresses low levels of antioxidant and anti‐electrophile protective enzymes, rendering it particularly vulnerable to free radical damage (Singh et al., 2015). Age‐associated mitochondrial dysfunction is an important part of aging. These energy powerhouses are capable of self‐replication but become dysfunctional with age through a variety of mechanisms, including a continuous vicious cycle of oxidative/electrophilic stress. To further establish the role of SFN in mitochondrial function and morphology during aging, we assessed mitochondrial ultrastructure, key electron transport chain complex protein expression, and markers of oxidative stress in the mouse hearts and mitochondrial respiration complexes in heart and SKM. Increased oxidants and electrophiles, leading to Nrf2 activity and Nrf2 responsive genes during aging, modify several mitochondrial proteins involved in energy metabolism and cause cristae disarrangement. Data obtained from our high‐resolution respirometry study on heart and SKM biopsies indicate that aging compromised functions of complex I, I&II, and maximal respiration, but this effect was more pronounced in heart. Mitochondrial complex activities heart of SFN‐fed old mice were fully restored close to the levels in the young control group of animals. Since these activities were severely lower in heart biopsies of old animals, the recovery appeared of significance. Increased CORE‐2 and NDUFS3 expression, mitochondrial cristae disarrangement, partial cristolysis, electron‐lucent matrix, and the reduced level of nitrotyrosine protein adducts following SFN fed to old animals likely also contributed to improved mitochondrial function and point to a protective role for SFN via activation of Nrf2‐dependent cellular defenses. We suggest that pharmacological activation of Nrf2 by SFN may remove excess ROS produced during aging by enhancing phase 2 detoxification and antioxidant enzyme activities and preserve electron transport chain (ETC) complex function. Since its effects on mitochondrial function were less pronounced in SKM where there are fewer mitochondria, SFN may exert other additional effects on SKM.

Cumulative oxidative damage in cells is an essential feature of cellular senescence and aging. Age‐associated oxidative stress promotes myocardial fibrosis, progressive increases in left ventricular stiffness, and dysfunction in heart, and may be one of the contributing factors to muscle weakness and sarcopenia. Age‐associated Nrf2‐dysfunction that can reduce antioxidant and anti‐electrophile enzyme activities, as well as mitochondrial function, contributes to the dysregulation of homeostasis and impairs the repair and regeneration capacity of the cell. We have previously demonstrated *in vitro* and *in vivo* that SFN protects and enhances Nrf2 signaling, consequently activating cytoprotective genes to counter radiation and doxorubicin‐induced cardiac toxicity and other related conditions (Chapple et al., 2012). We have recently demonstrated that SFN enhanced Nrf2 signaling and cytoprotective gene activity in mouse heart that confers protection from oxidative damage (Singh et al., 2015).

In the present study, we examined whether SFN in the diet stimulates Nrf2 activity and responsive genes in two most important functional tissues, SKM, and heart. We determined the amount of active nuclear Nrf2 in the SKM and heart of young and old mice fed SFN or control diet. SKM and heart of SFN diet fed young and old mice showed a significant increase in ARE‐binding activity, countering the significantly reduced activity observed in old mice on the control diet. The drop in Nrf2 activity in the old control group of mice clearly indicated an inability of the SKM and heart, which already express low levels of protective enzymes, to protect themselves from oxidative and electrophilic assault and function normally. Thus, in this study, we have shown for the first time that increased oxidative stress in the aging heart and SKM correlates with failure of protective response due to dysregulation of Nrf2 and that this process can be attenuated by the administration of SFN to old (21‐ to 22‐month‐old) mice.

Transcript levels of genes responsible for expression of antioxidant, anti‐electrophile, and glutathione synthesis pathway enzymes (A) in heart and (B) in SKM of control and SFN diet fed mice were examined. (A) In heart, *Catalase*, *Sod1*,*Sod2*, and *Nrf2* showed the best correlation with decreased function in old mice and restoration by SFN. In addition, *Akr3* was increased in old mice and this increase was reduced by SFN. *Sod1* and *Sod2* protect the mitochondrial complexes and active enzymes in the mitochondrial matrix and permit the maintenance of homeostasis and mitochondrial integrity (Piao et al., 2010). (B) In SKM, there were just as many suspects: *Catalase*,*Sod 1*,*Gpx1*,*Gsta4*, and *Nrf2*. It should come as no surprise that there is only partial overlap between genes affected in heart and SKM, particularly in view of the lesser effect of aging and SFN on mitochondria in SKM. *Catalase* and *Sod1* might exert some of their action at the level of mitochondria but also increase the level of antioxidant and anti‐electrophilic defense mechanisms and contribute to the initial lower lipid peroxidation products and 4‐HNE concentration, to restore function in the SKM of old mice. Increased capacity for enzymatic scavenging of superoxide radical could be a major protective adaptation against free radical damage in muscle and may therefore be a major protection against the development or onset of sarcopenia, and *Gpx1* and *Gsta4* might additionally be involved. Our findings demonstrate that increased active Nrf2 in SKM and heart facilitates transcriptional activation of genes expressing antioxidants and anti‐electrophile enzymes which can play a critical role during aging. Thus, SFN could effectively prevent heart and SKM tissue dysfunction by restoring multiple cellular defenses through other activities of the Keap1/Nrf2 pathway, such as autophagy, glutathione biosynthesis, and mitochondrial biogenesis during aging and aging‐related diseases.

In conclusion, age‐associated myopathy is an intrinsic process with profound cellular and molecular changes that results in impaired cardiac and SKM function. Using a mouse aging model, we demonstrated that SFN diet can restore SKM and heart functions in the aged mice. Our studies did not find any adverse effects of SFN containing diet on the mice. The protection of respiratory chain complex activity, increased mitochondrial complex proteins, and lowering of oxidative damage by SFN in the aging heart suggest that SFN enhances mitochondrial function. Further analysis of pathways activated by *Gpx1* and *Gsta4* warrant attention because dissection of the involved mechanisms could lead to new therapies for sarcopenia. Protecting and enhancing Nrf2‐driven biological functions may prove to be a safe and effective strategy to counter the loss of SKM and heart functions in older adults. Our findings provide a mechanistic understanding and information about novel biomarkers and surrogate endpoints, which could be applied in clinical settings.

## CONFLICT OF INTEREST

All authors declare no competing interests.

## AUTHOR CONTRIBUTIONS

S.P.S. conceptualized, obtained funding, designed the studies, performed experiments, analyzed data, and performed bioinformatics analyses for the project. C.B., I.A., P.S., and E.C. designed and carried out experiments and contributed to manuscript preparation. P.T.P., E.B., S. J., A.C., M.B., and S.A. contributed to manuscript preparation and analyses of literature. S.J., M.B., and C.B. analyzed data and contributed to manuscript. S.P.S., C.B., E.C., and I.A. analyzed and validated data, and wrote the manuscript. All authors read and approved the final manuscript.

## Data Availability

Data are available upon request. The majority of results corresponding to the current study are included in the article or uploaded as supplementary information. Other data are available on request from the authors.
