# Supplementary material for: Sulforaphane prevents age‐associated cardiac and muscular dysfunction through Nrf2 signaling
Source: Aging Cell. 2020 Oct 17;19(11):e13261. doi: 10.1111/acel.13261 (PMC7681049; doi:10.1111/acel.13261)
Supplement: Supplementary file 1 [file ACEL-19-e13261-s001.pptx]

## Slide 1
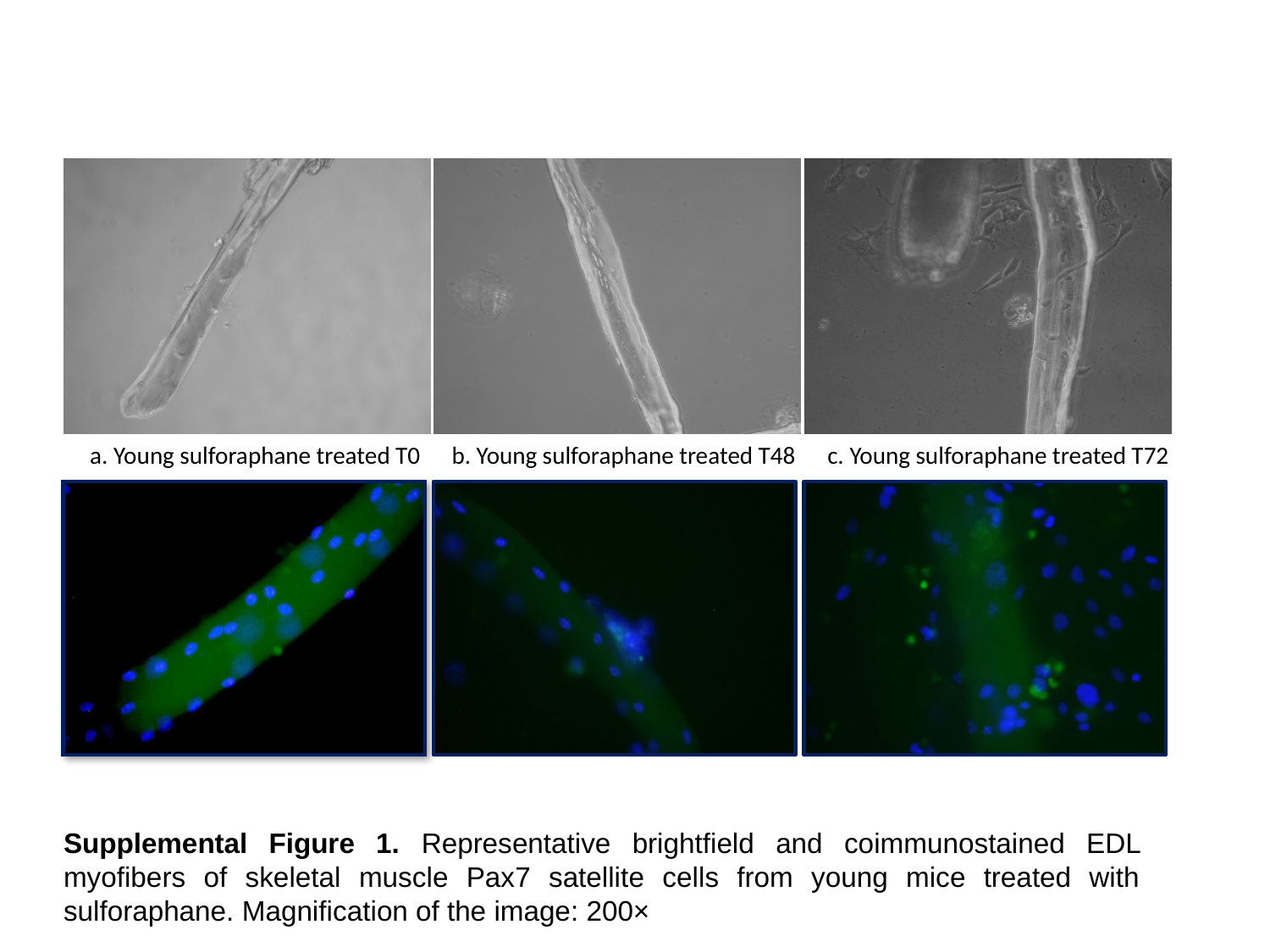

a. Young sulforaphane treated T0
b. Young sulforaphane treated T48
c. Young sulforaphane treated T72
Supplemental Figure 1. Representative brightfield and coimmunostained EDL myofibers of skeletal muscle Pax7 satellite cells from young mice treated with sulforaphane. Magnification of the image: 200×

## Slide 2
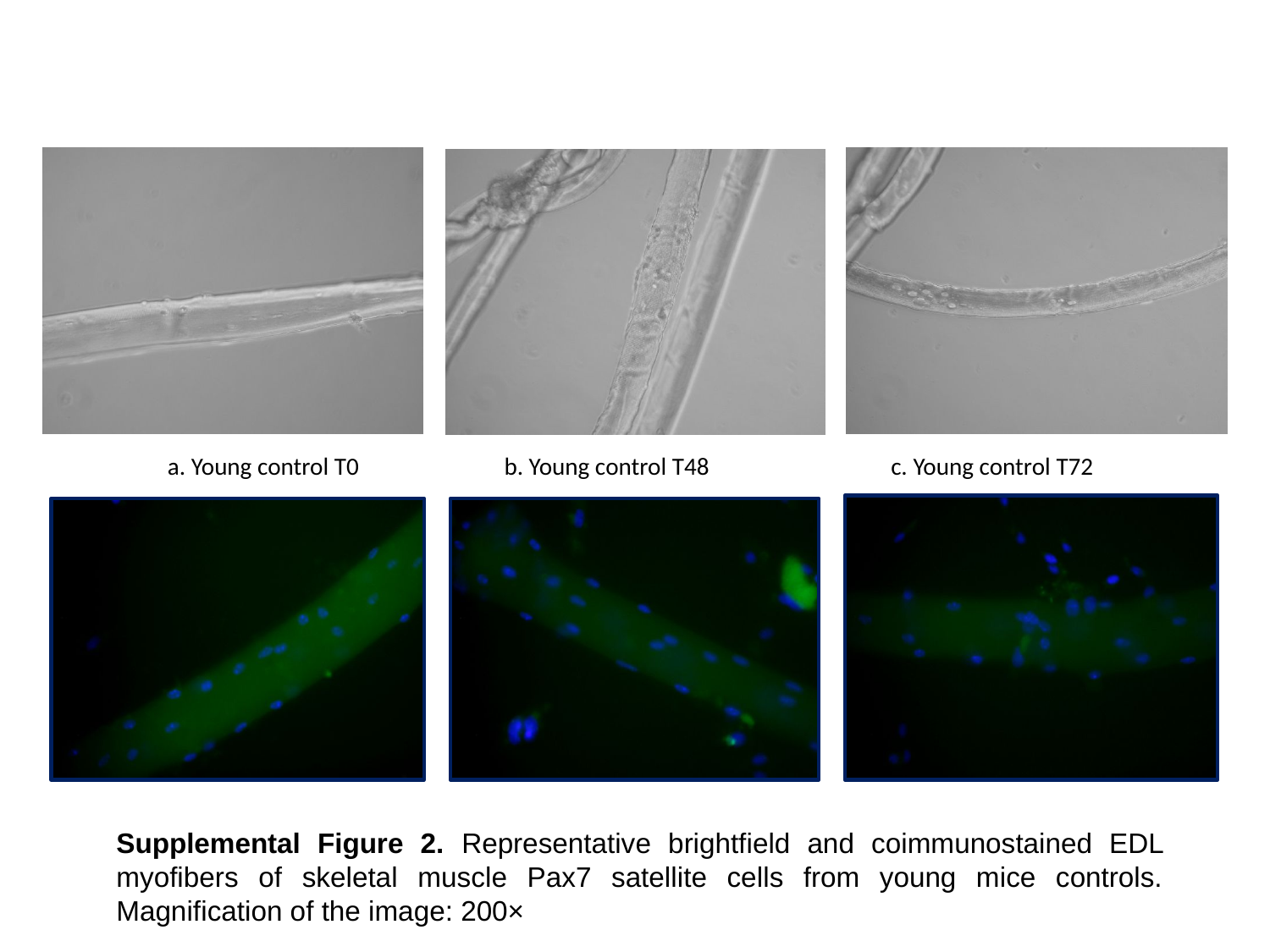

a. Young control T0
c. Young control T72
b. Young control T48
Supplemental Figure 2. Representative brightfield and coimmunostained EDL myofibers of skeletal muscle Pax7 satellite cells from young mice controls. Magnification of the image: 200×

## Slide 3
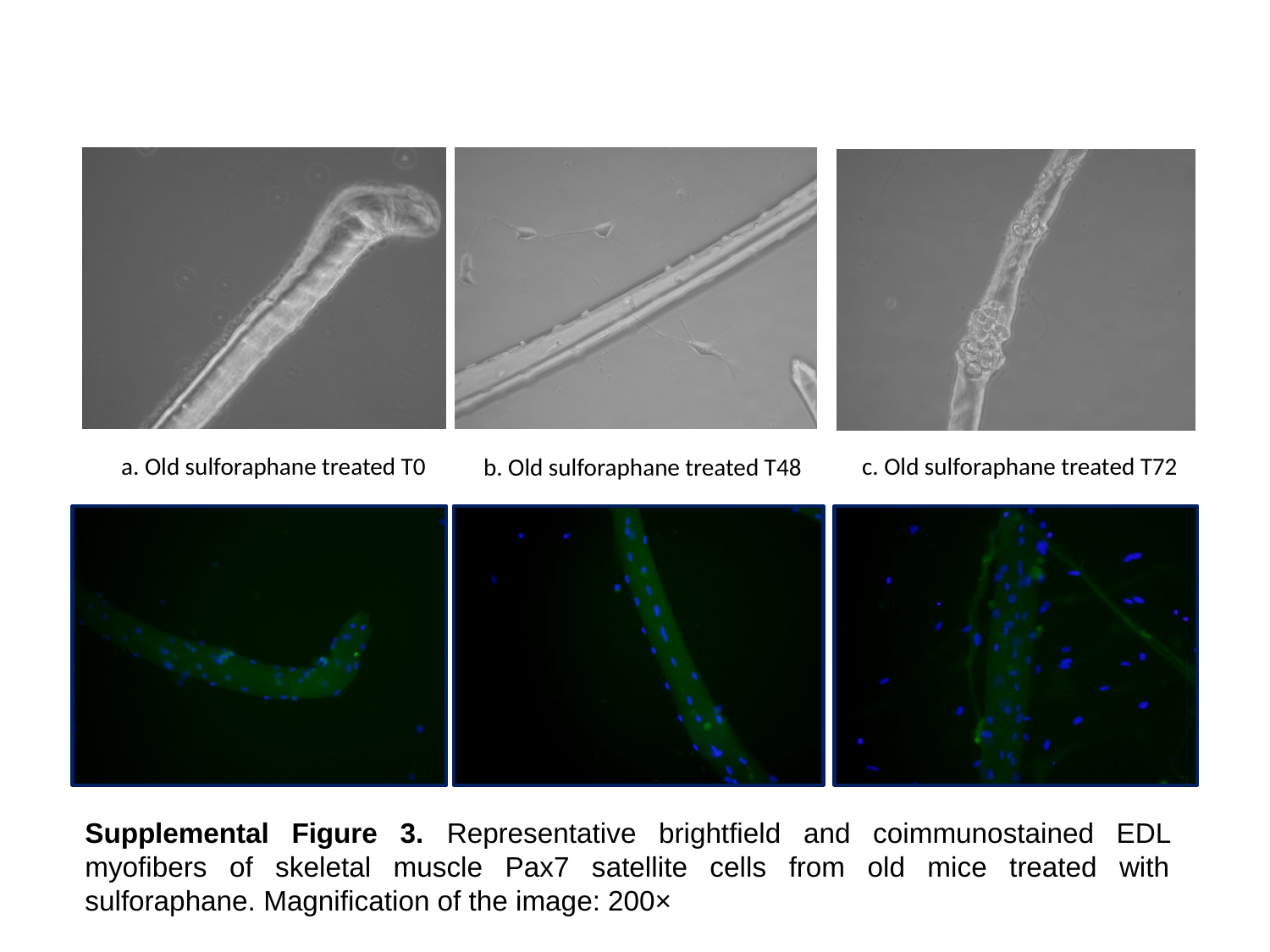

a. Old sulforaphane treated T0
c. Old sulforaphane treated T72
b. Old sulforaphane treated T48
Supplemental Figure 3. Representative brightfield and coimmunostained EDL myofibers of skeletal muscle Pax7 satellite cells from old mice treated with sulforaphane. Magnification of the image: 200×

## Slide 4
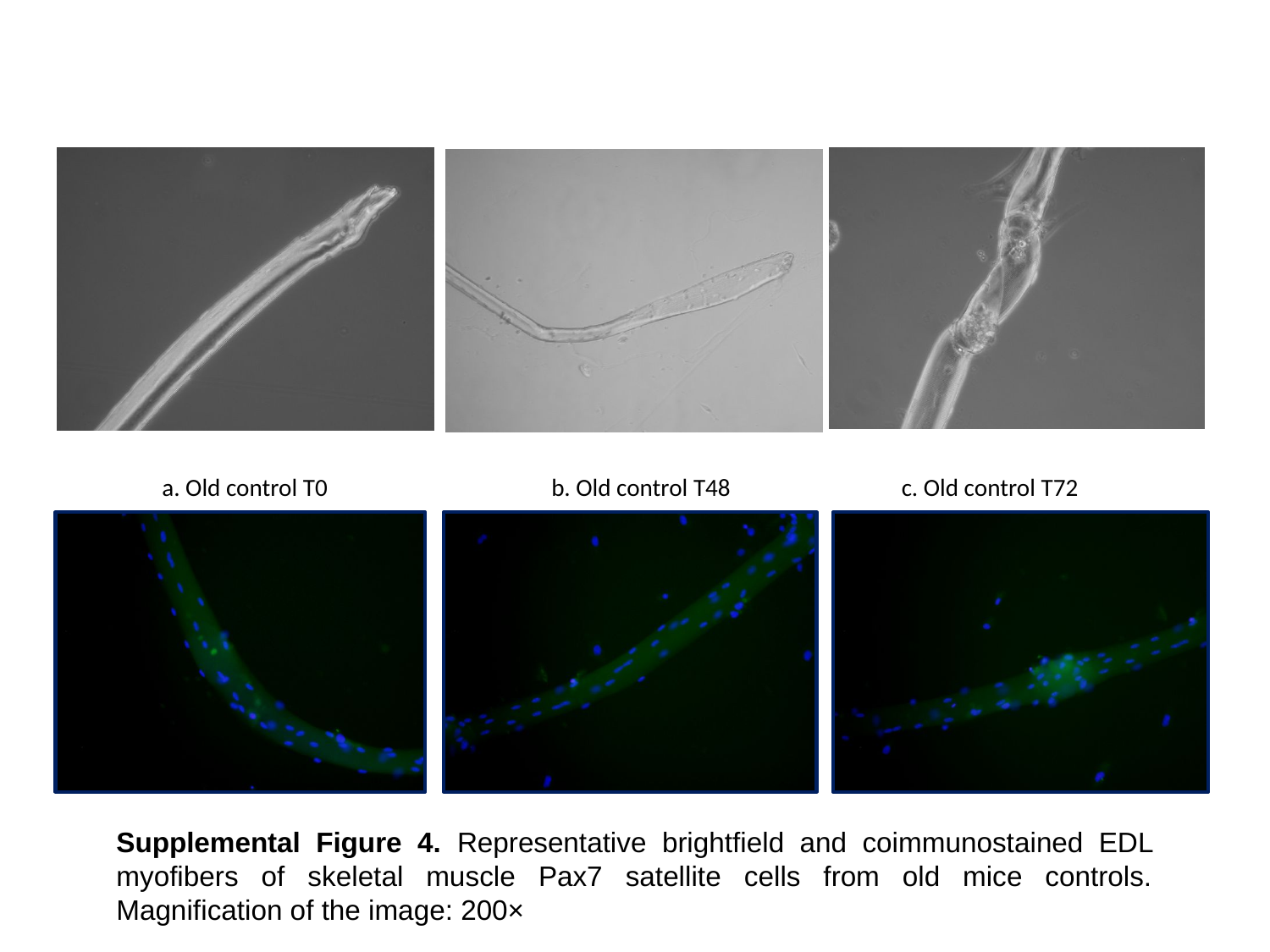

a. Old control T0
b. Old control T48
c. Old control T72
Supplemental Figure 4. Representative brightfield and coimmunostained EDL myofibers of skeletal muscle Pax7 satellite cells from old mice controls. Magnification of the image: 200×

## Slide 5
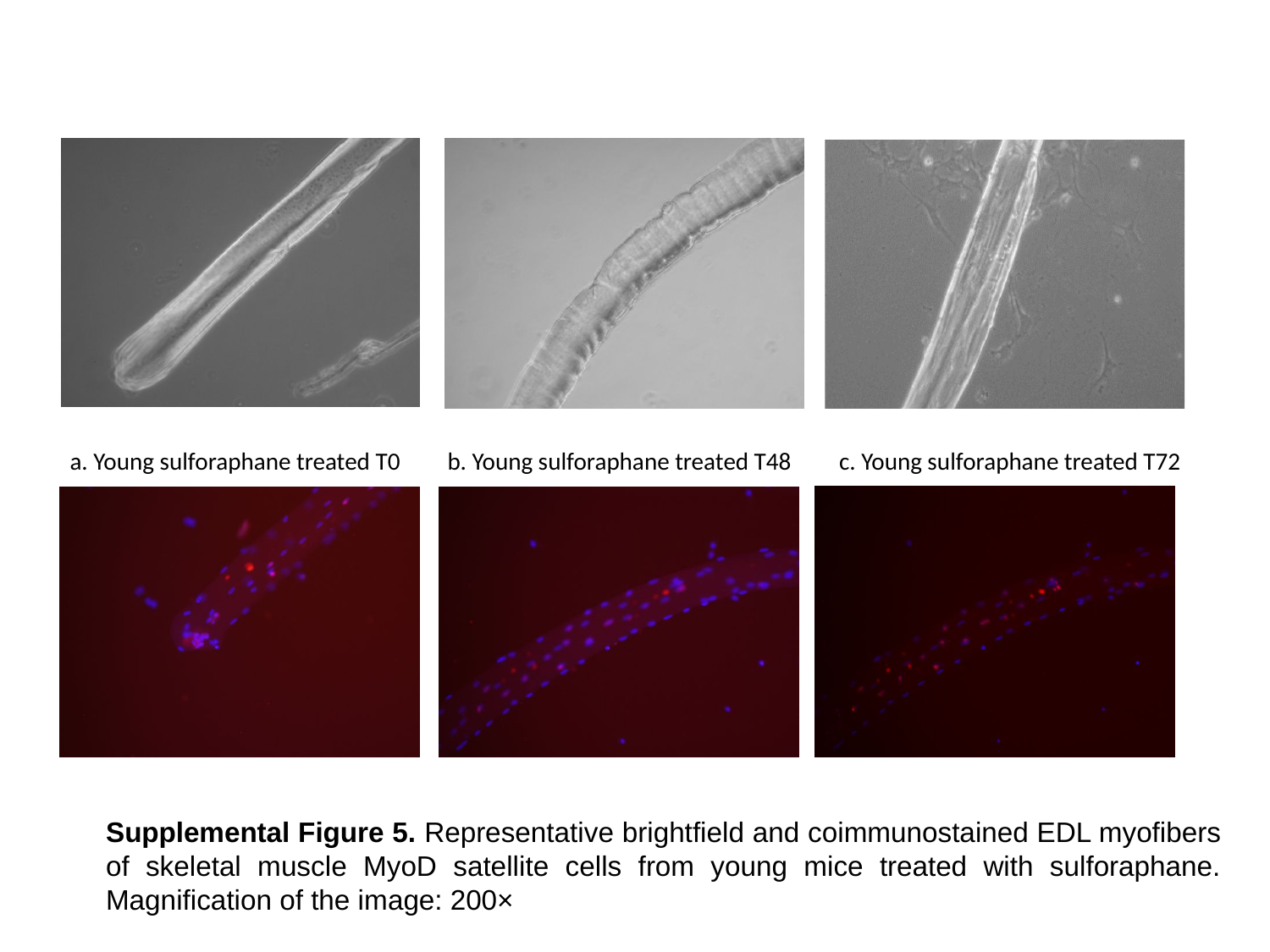

a. Young sulforaphane treated T0
b. Young sulforaphane treated T48
c. Young sulforaphane treated T72
Supplemental Figure 5. Representative brightfield and coimmunostained EDL myofibers of skeletal muscle MyoD satellite cells from young mice treated with sulforaphane. Magnification of the image: 200×

## Slide 6
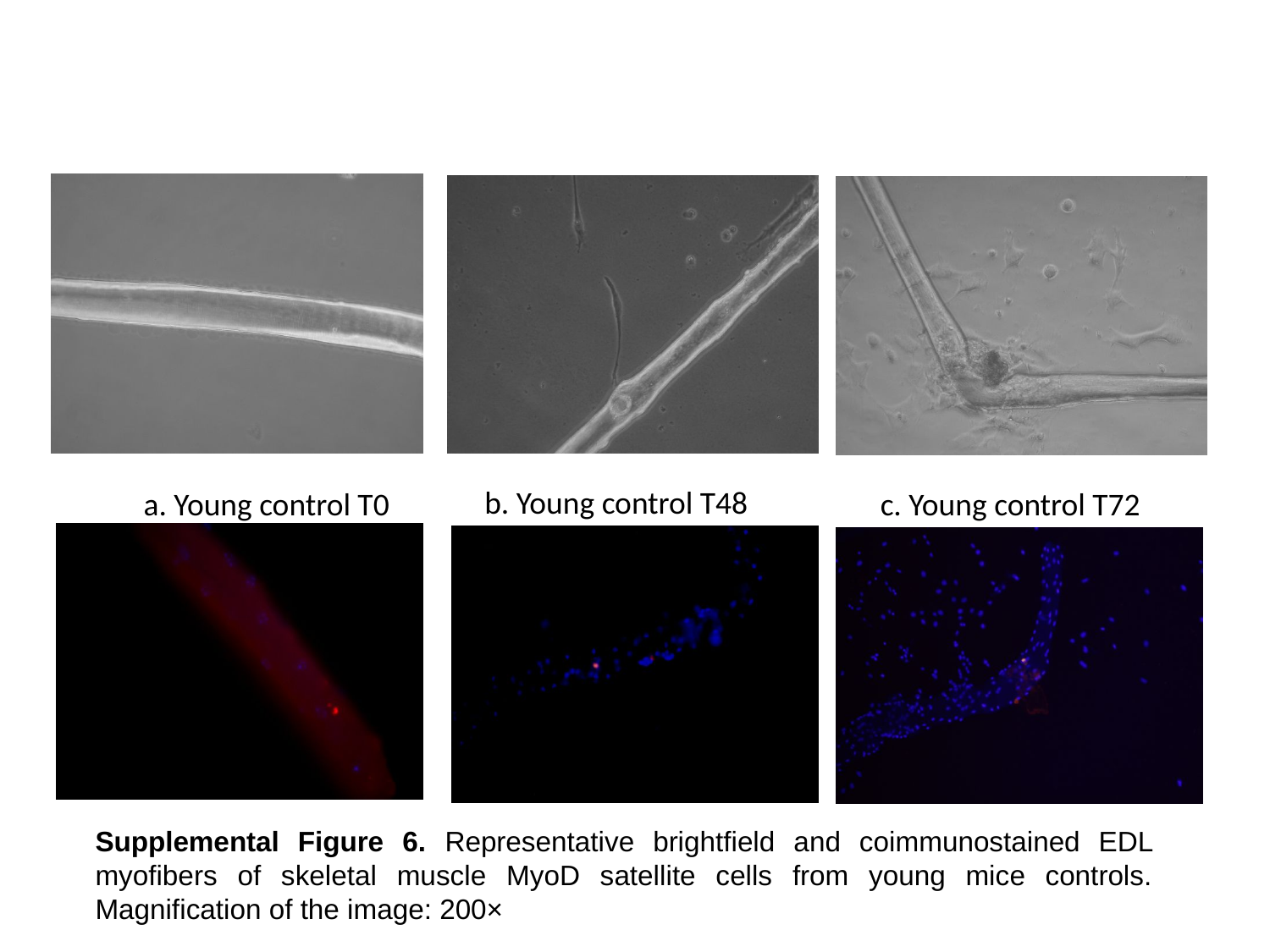

b. Young control T48
a. Young control T0
c. Young control T72
Supplemental Figure 6. Representative brightfield and coimmunostained EDL myofibers of skeletal muscle MyoD satellite cells from young mice controls. Magnification of the image: 200×

## Slide 7
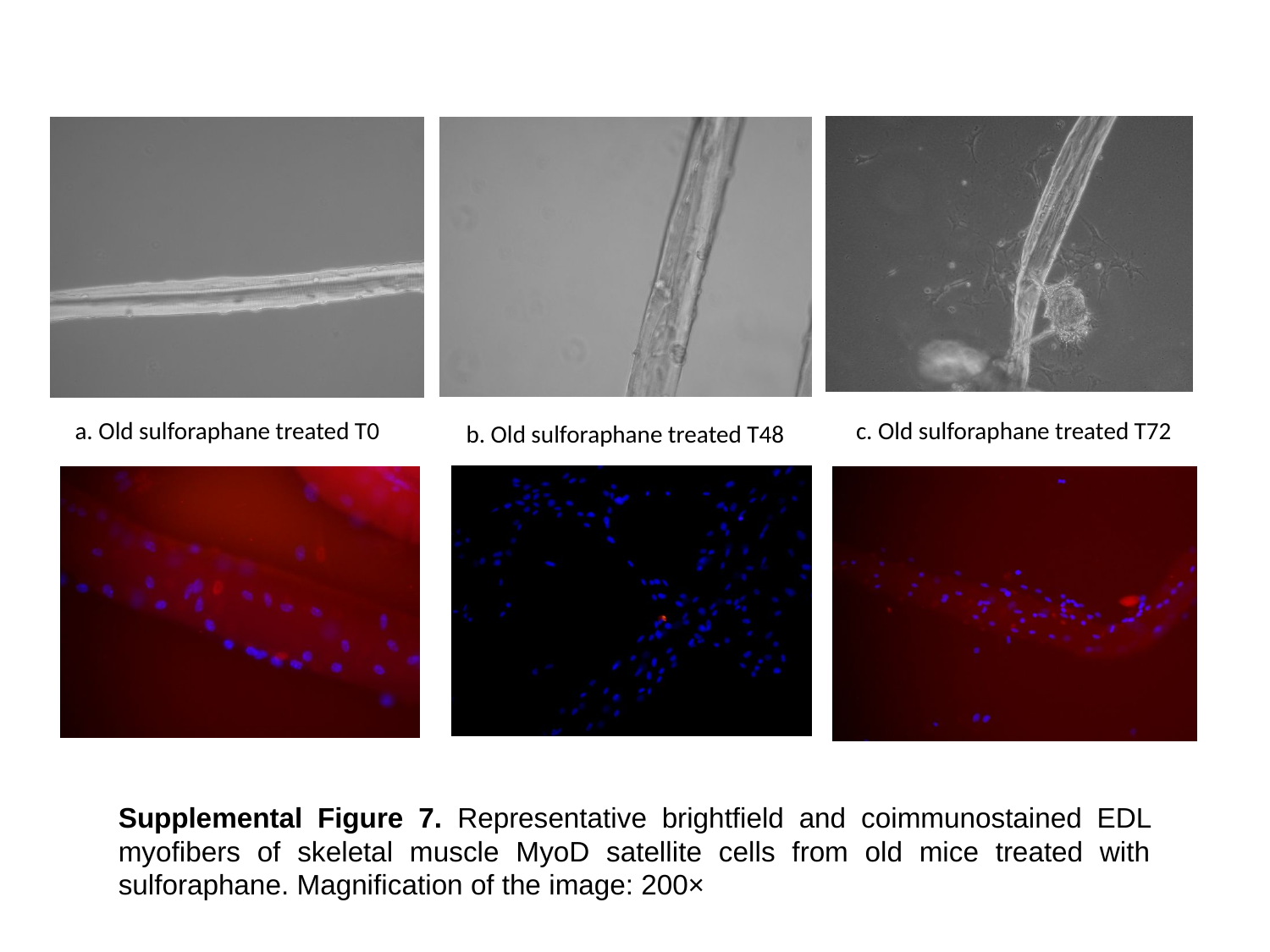

a. Old sulforaphane treated T0
c. Old sulforaphane treated T72
b. Old sulforaphane treated T48
Supplemental Figure 7. Representative brightfield and coimmunostained EDL myofibers of skeletal muscle MyoD satellite cells from old mice treated with sulforaphane. Magnification of the image: 200×

## Slide 8
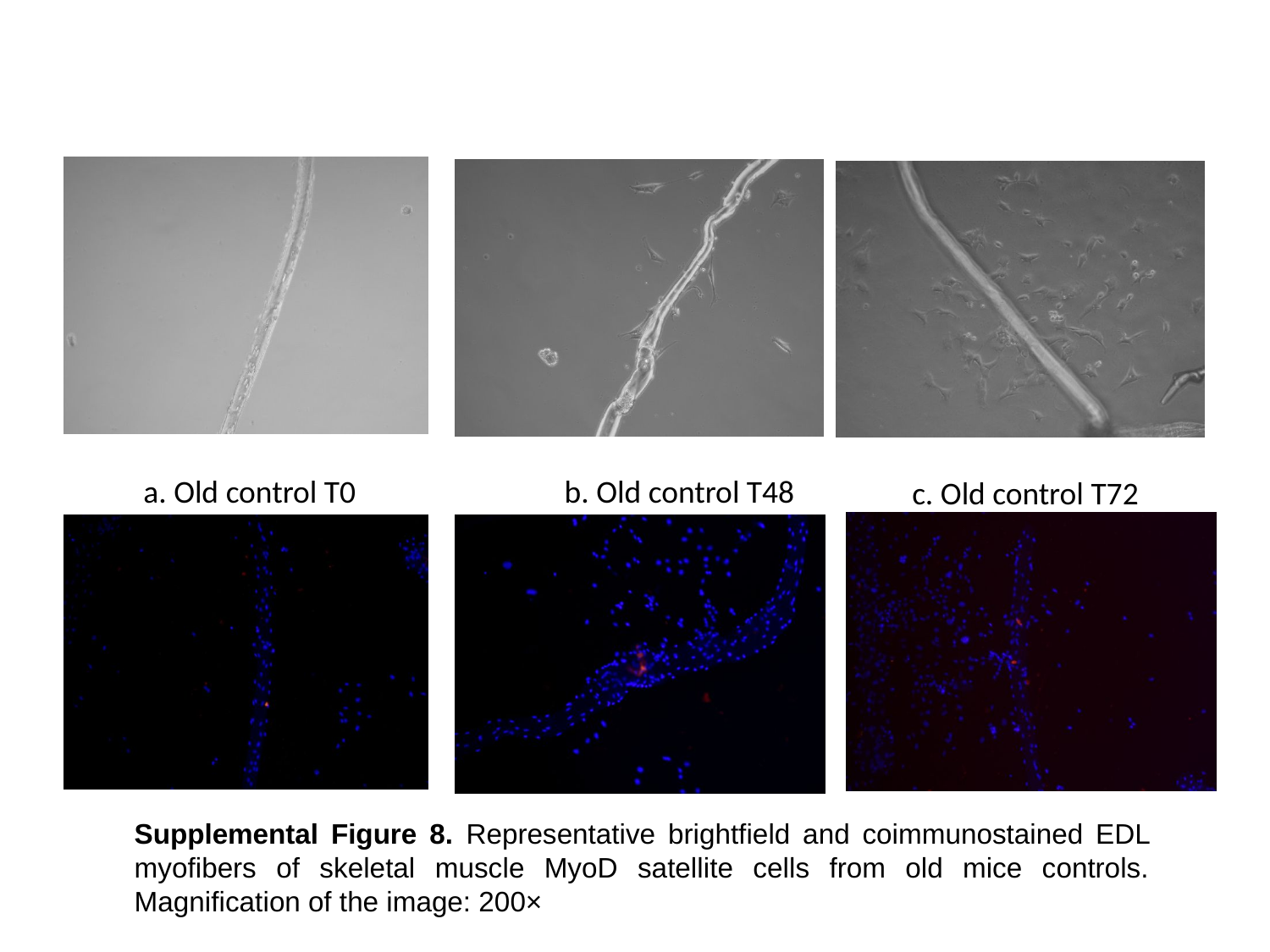

a. Old control T0
b. Old control T48
c. Old control T72
Supplemental Figure 8. Representative brightfield and coimmunostained EDL myofibers of skeletal muscle MyoD satellite cells from old mice controls. Magnification of the image: 200×

## Slide 9
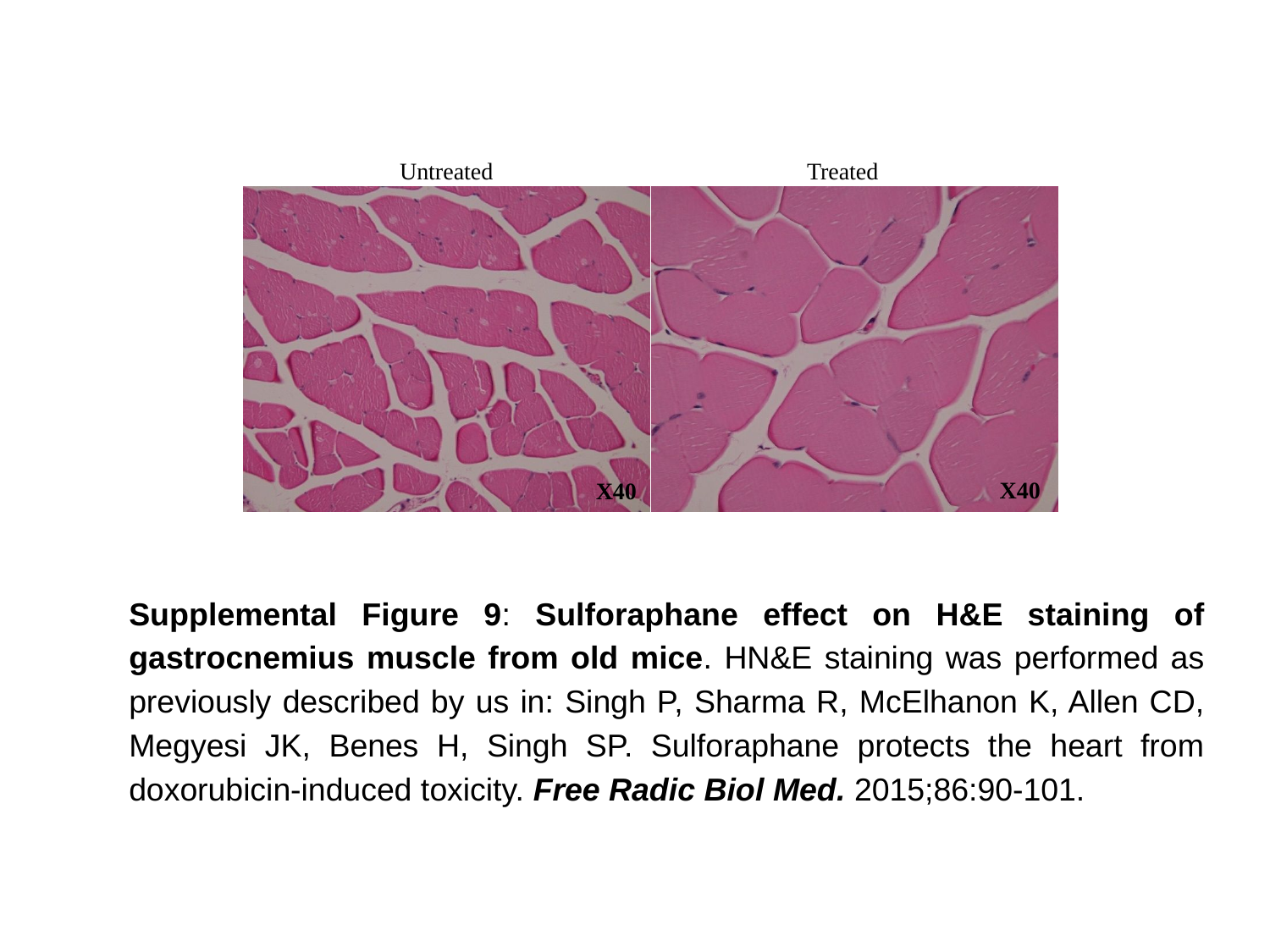

Untreated
Treated
X40
X40
Supplemental Figure 9: Sulforaphane effect on H&E staining of gastrocnemius muscle from old mice. HN&E staining was performed as previously described by us in: Singh P, Sharma R, McElhanon K, Allen CD, Megyesi JK, Benes H, Singh SP. Sulforaphane protects the heart from doxorubicin-induced toxicity. Free Radic Biol Med. 2015;86:90-101.

## Slide 10
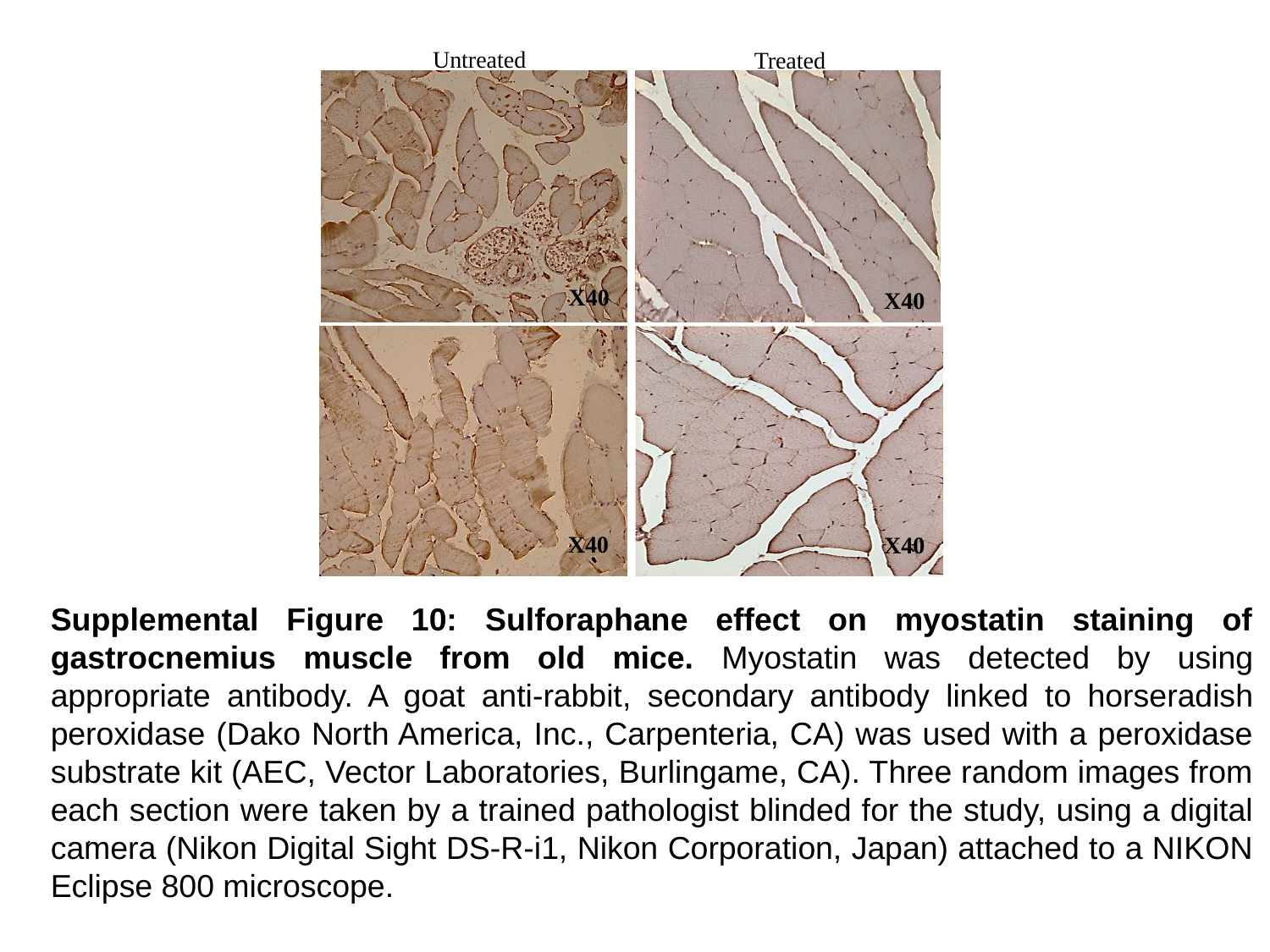

Untreated
Treated
X40
X40
X40
X40
Supplemental Figure 10: Sulforaphane effect on myostatin staining of gastrocnemius muscle from old mice. Myostatin was detected by using appropriate antibody. A goat anti-rabbit, secondary antibody linked to horseradish peroxidase (Dako North America, Inc., Carpenteria, CA) was used with a peroxidase substrate kit (AEC, Vector Laboratories, Burlingame, CA). Three random images from each section were taken by a trained pathologist blinded for the study, using a digital camera (Nikon Digital Sight DS-R-i1, Nikon Corporation, Japan) attached to a NIKON Eclipse 800 microscope.
.

## Slide 11
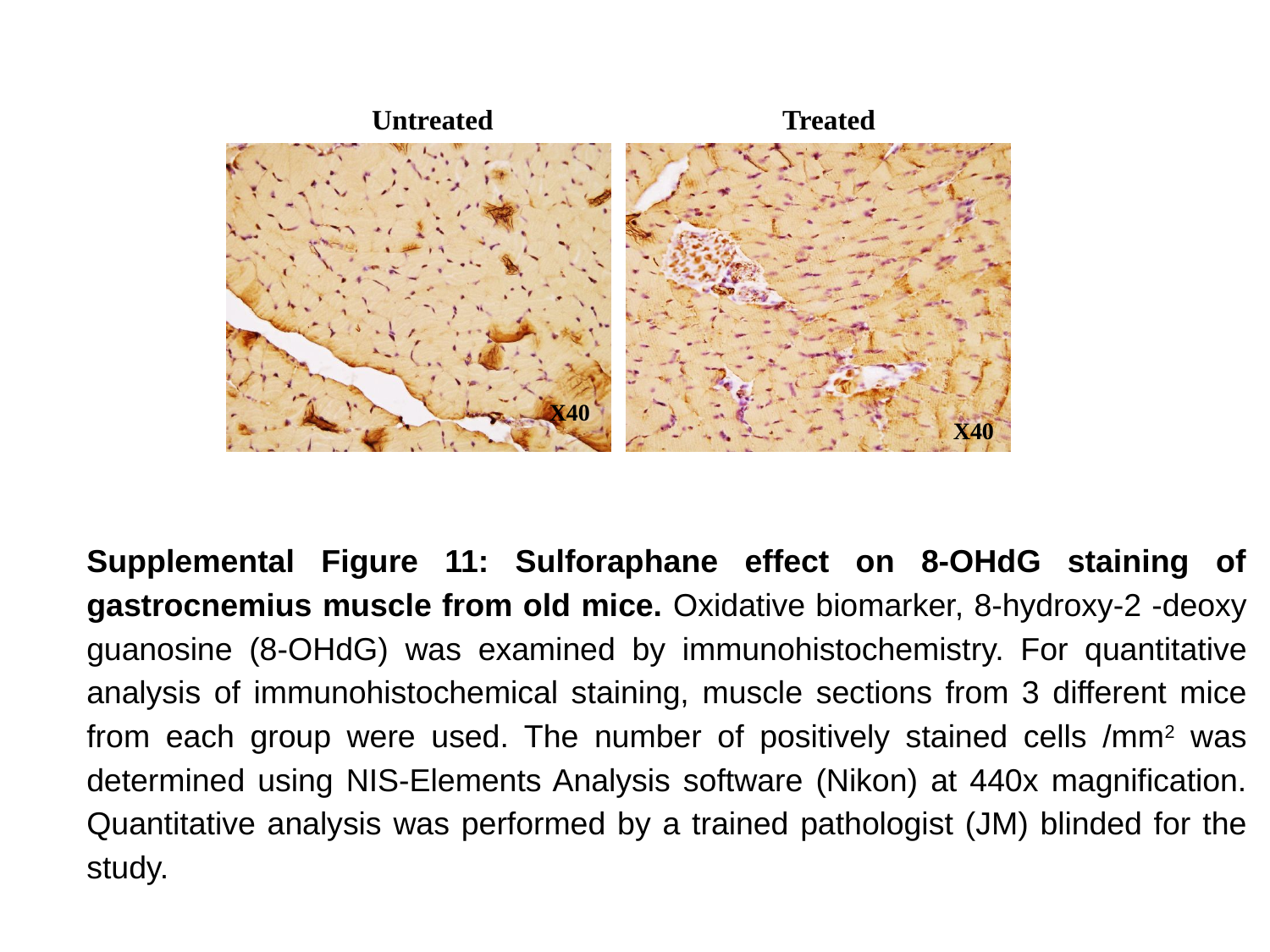

Untreated
Treated
X40
X40
Supplemental Figure 11: Sulforaphane effect on 8-OHdG staining of gastrocnemius muscle from old mice. Oxidative biomarker, 8-hydroxy-2 -deoxy guanosine (8-OHdG) was examined by immunohistochemistry. For quantitative analysis of immunohistochemical staining, muscle sections from 3 different mice from each group were used. The number of positively stained cells /mm2 was determined using NIS-Elements Analysis software (Nikon) at 440x magnification. Quantitative analysis was performed by a trained pathologist (JM) blinded for the study.

## Slide 12
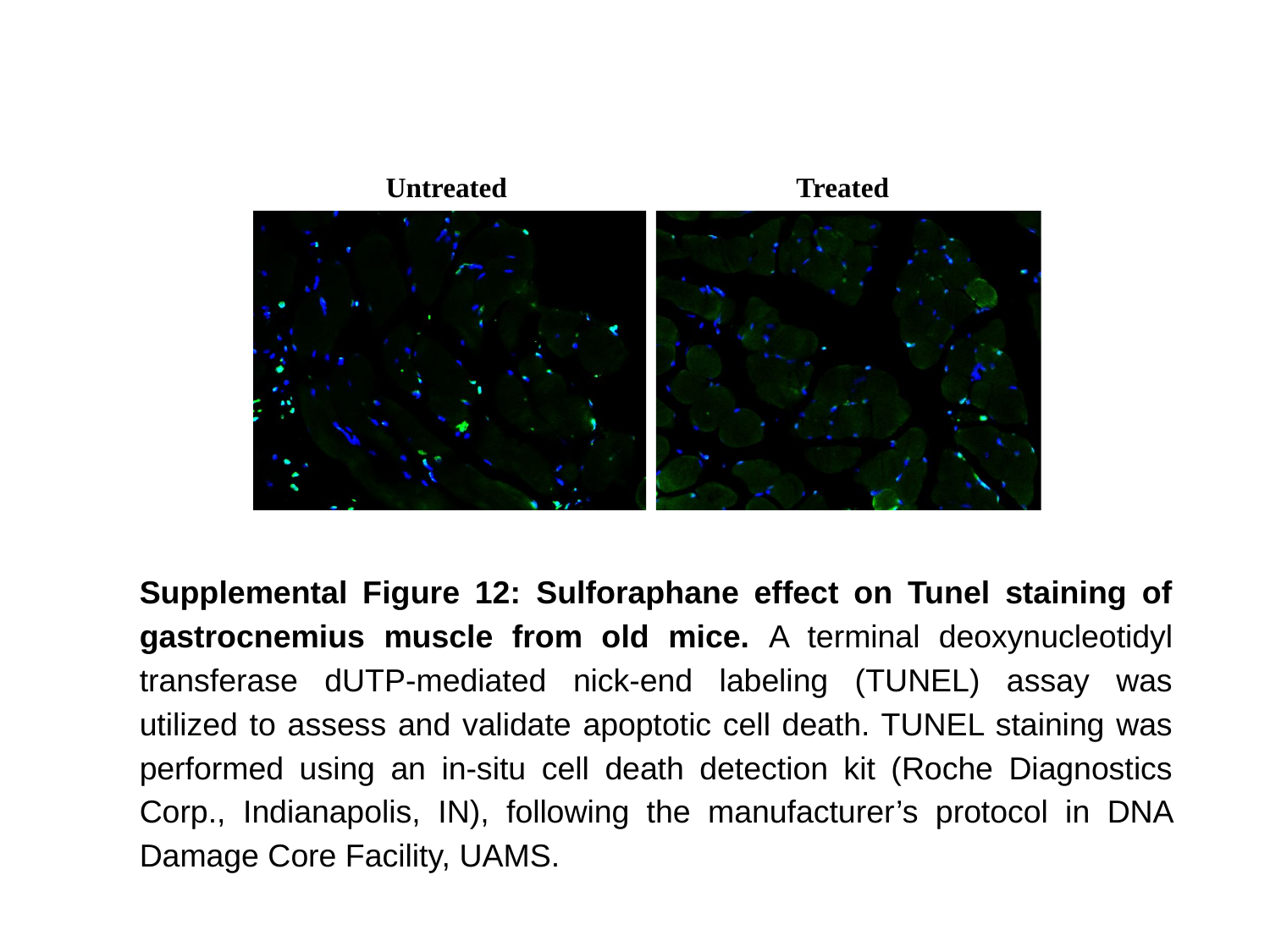

Untreated
Treated
Supplemental Figure 12: Sulforaphane effect on Tunel staining of gastrocnemius muscle from old mice. A terminal deoxynucleotidyl transferase dUTP-mediated nick-end labeling (TUNEL) assay was utilized to assess and validate apoptotic cell death. TUNEL staining was performed using an in-situ cell death detection kit (Roche Diagnostics Corp., Indianapolis, IN), following the manufacturer’s protocol in DNA Damage Core Facility, UAMS.

## Slide 13
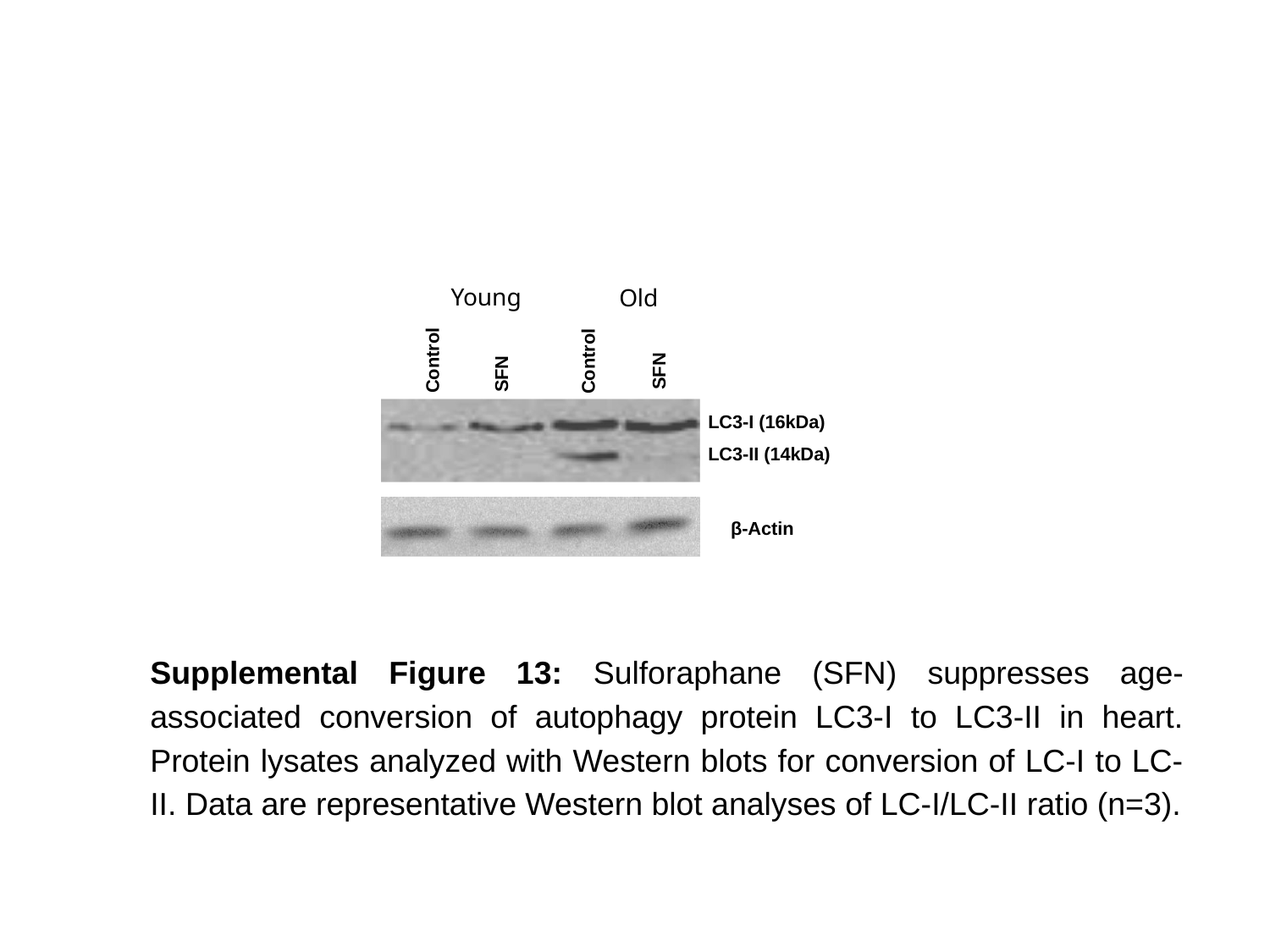

Young
Old
Control
Control
SFN
SFN
LC3-I (16kDa)
LC3-II (14kDa)
β-Actin
Supplemental Figure 13: Sulforaphane (SFN) suppresses age-associated conversion of autophagy protein LC3-I to LC3-II in heart. Protein lysates analyzed with Western blots for conversion of LC-I to LC-II. Data are representative Western blot analyses of LC-I/LC-II ratio (n=3).
