# Supplementary material for: Sulforaphane prevents age‐associated cardiac and muscular dysfunction through Nrf2 signaling
Source: Aging Cell. 2020 Oct 17;19(11):e13261. doi: 10.1111/acel.13261 (PMC7681049; doi:10.1111/acel.13261)
Supplement: Supplementary file 2 [file ACEL-19-e13261-s002.docx]

**Supplemental Table 1**. **Complete blood count (CBC) of young and old mice fed with control or SFN diet**

| **Hematological Parameters** | **Pre-treatment** | | **Post-treatment** | | | | |
| --- | --- | --- | --- | --- | --- | --- | --- |
|  | **Young** | **Old** | **Young Control** | **Young+SFN** | **Old Control** | **Old+SFN** |  |
| **WBC (K/μl)** | **12.65±1.24** | **11.51±2.04** | **12.08±0.88** | **12.82±1.66** | **10.39±0.78** | **11.34±3.15** |  |
| **NE (K/μl)** | **2.22±0.07** | **2.42±0.10** | **2.34±0.02** | **2.61±0.27** | **2.35±0.02** | **2.59±1.43** |  |
| **LY (K/μl)** | **9.04±0.95** | **10.14±1.15** | **8.17±0.94** | **10.50±1.34** | **8.27±0.68** | **8.75±3.03** |  |
| **MO (K/μl)** | **0.75±0.13** | **0.82±0.11** | **0.84±0.07** | **0.86±0.11** | **0.73±0.09** | **0.75±0.35** |  |
| **EO (K/μl)** | **0.48±0.09** | **0.51±0.07** | **0.44±0.02** | **0.44±0.02** | **0.44±0.02** | **0.42±0.20** |  |
| **BA (K/μl)** | **0.17±0.03** | **0.18±0.04** | **0.15±0.15** | **0.16±0.01** | **0.15±0.12** | **0.14±0.04** |  |
|  |  |  |  |  |  |  |  |
| **NE (%)** | **17.17±0.42** | **16.34±0.32** | **16.75±0.52** | **15.99±1.42** | **14.81±2.37** | **18.58±17.28** |  |
| **LY (%)** | **72.12±1.67** | **73.12±1.87** | **89.84±1.66** | **88.98±1.12** | **84.84±1.13** | **79.11±22.93** |  |
| **MO (%)** | **5.72±0.62** | **5.92±0.67** | **5.95±1.11** | **5.58±0.76** | **5.90±0.77** | **5.60±4.83** |  |
| **EO (%)** | **3.73±0.66** | **3.81±0.69** | **3.40±0.19** | **3.32±0.23** | **3.32±0.25** | **3.30±1.90** |  |
| **BA (%)** | **1.30±0.32** | **1.40±0.42** | **1.06±0.02** | **1.12±0.05** | **1.13±0.04** | **1.43±0.22** |  |
|  |  |  |  |  |  |  |  |
| **RBC (M/μl)** | **9.32±0.05** | **8.82±0.15** | **10.00±0.26** | **9.29±0.52** | **8.56±0.22** | **8.97±0.12** |  |
| **Hb (g/dL)** | **12.83±0.37** | **11.93±0.42** | **13.73±0.13** | **12.40±0.50** | **11.17±0.31** | **12.48±0.70** |  |
| **HCT (%)** | **50.20±1.01** | **47.20±2.10** | **58.15±2.36** | **52.50±1.85** | **47.43±1.94** | **48.03±5.60** |  |
| **MCV (FL)** | **53.97±1.58** | **51.87±2.18** | **58.03±1.37** | **56.58±1.68** | **55.43±0.91** | **52.65±1.11** |  |
| **MCH (pg)** | **13.79±0.07** | **12.99±0.08** | **13.75±0.26** | **13.35±0.41** | **13.03±0.91** | **13.68±2.10** |  |
| **MCHC (g/dL)** | **25.57±0.67** | **23.96±0.54** | **23.63±0.90** | **23.60±0.24** | **23.57±0.47** | **26.05±2.10** |  |
| **RDW (%)** | **21.29±3.70** | **20.92±4.00** | **17.75±0.65** | **18.58±0.60** | **18.47±0.64** | **20.33±2.91** |  |

The blood samples of each mice were used for determination of hematological parameters using the Hemavet instrument (Drew Scientific, Dallas, TX). Statistically difference (n=5) using Tukey's multiple comparisons test between control and SFN (young or old) and group of animals suggest no significant differences in all comparisons. **Abbreviations:** WBC (White blood cells), NE (Neutrophils), LY (Lymphocytes), MO (Monocytes), EO (Eosinophils), BA (Basophils), RBC (Red blood cells), Hb (Hemoglobin), HCT (Hematocrit), MCV (Mean corpuscular volume), MCH (Mean Corpuscular Hemoglobin), MCHC (Mean Corpuscular Hemoglobin Concentration), RDW (Red cell distribution width), K/µl (thousands per milliliter), M/μl (million per microliter), FL (femoliter), g/dL (grams per deciliter), pg (picogram).
